# Formation of a complex between TMEM217 and the sodium-proton exchanger SLC9C1 is crucial for mouse sperm motility and male fertility

**DOI:** 10.1073/pnas.2513924122

**Published:** 2025-10-15

**Authors:** Rie Iida-Norita, Haruhiko Miyata, Akinori Ninomiya, Chihiro Emori, Maki Kamoshita, Chen Pan, Haoting Wang, Masahito Ikawa

**Affiliations:** ^a^Department of Experimental Genome Research, Research Institute for Microbial Diseases, The University of Osaka, Suita, Osaka 565-0871, Japan; ^b^Animal Resource Center for Infectious Diseases, Immunology Frontier Research Center, The University of Osaka, Suita, Osaka 565-0871, Japan; ^c^Laboratory of Experimental Genome Research, Graduate School of Pharmaceutical Sciences, The University of Osaka, Suita, Osaka 565-0871, Japan; ^d^Division of Microbiology and Immunology, Center for Infectious Disease Education and Research, The University of Osaka, Suita, Osaka 565-0871, Japan; ^e^Team of Vaccine Evaluation, Center for Advanced Modalities and DDS, The University of Osaka, Suita, Osaka 565-0871, Japan; ^f^Laboratory of Reproductive Systems Biology, The Institute of Medical Science, The University of Tokyo, Tokyo 108-8639, Japan

**Keywords:** sodium-proton exchanger, sperm motility, male fertility, cAMP signaling, therapy for infertility

## Abstract

Sperm motility is essential for male fertility and is tightly controlled by signaling events in the flagellum. *Slc9c1* encodes a sperm-specific Na^+^/H^+^ exchanger (sNHE/SLC9C1) that localizes to the flagellum and is indispensable for sperm motility and male fertility. SLC9C1 is unique among Na^+^/H^+^ exchangers in that it possesses a voltage-sensing domain (VSD), the physiological function of which remains poorly understood in mammals. Here, by analyzing coevolving genes with *Slc9c1*, we identified *Tmem217*, which encodes a transmembrane protein that is localized in the sperm flagellum. Knockout (KO) of *Tmem217* in mice resulted in sperm motility defects and male infertility, phenocopying *Slc9c1* KO mice. Coimmunoprecipitation and structural prediction analyses indicated that TMEM217 binds to SLC9C1 via its VSD. Further analyses indicated that the amounts of SLC9C1 and its associated protein, soluble adenylyl cyclase (sAC), were lost in mature *Tmem217* KO spermatozoa, leading to disrupted 3′,5′-cyclic monophosphate (cAMP) signaling pathways. Remarkably, cAMP analogs restored the impaired motility and fertilizing ability of *Tmem217* KO spermatozoa in vitro, validating the essential role of TMEM217 in regulating cAMP production. Our findings indicate that the association of TMEM217 with SLC9C1 via its VSD is critical for the proper organization and function of the SLC9C1–sAC–cAMP axis in mature spermatozoa.

## Significance

Asthenozoospermia, characterized by impaired sperm motility, is a common cause of male infertility. The Na^+^/H^+^ exchanger SLC9C1 is a critical regulator of sperm motility through the modulation of intracellular signaling. In this study, we demonstrate that TMEM217 is an interaction partner of SLC9C1. Our results indicate that the TMEM217–SLC9C1 complex is essential for 3′,5′-cyclic monophosphate (cAMP) signaling, sperm motility, and male fertility. Importantly, impaired sperm motility and fertilization ability of *Tmem217* Knockout (KO) spermatozoa were rescued by enhancing cAMP signaling. Our findings reveal the biological significance of the conserved TMEM217–SLC9C1 interaction and give insights into the diagnosis and therapeutic strategy of asthenozoospermia associated with defective cAMP signaling.

Male infertility is a global issue affecting individuals who desire to have children. Among various causes, asthenozoospermia, defined as sperm motility below 40% or progressive motility below 32%, accounts for approximately 19% of male infertility cases (1), which often arises from defects in flagellum structure, energy production, or signaling pathways. After ejaculation, sperm motility is crucial for navigating the female reproductive tract, reaching the oocyte in the oviductal ampulla, and penetrating through the zona pellucida surrounding the oocyte. During the transition through the female reproductive tract, changes in bicarbonate (HCO_3_^−^) concentration due to mixing with seminal plasma trigger the production of adenosine 3′,5′-cyclic monophosphate (cAMP) by soluble adenylyl cyclase (sAC/*ADCY10*), activating downstream protein kinase A (PKA) signaling pathways that drive sperm motility (2–9).

The Na^+^/H^+^ exchanger (NHE/SLC9) family, which regulates intracellular pH (pH_i_) through ion transport, includes 11 isoforms grouped into SLC9A, SLC9B, and SLC9C subfamilies (10–13). Among these, sperm-specific NHE (sNHE/SLC9C1) is localized to the principal piece of spermatozoa and plays a critical role in sperm motility (14). Unique to the NHE/SLC9 family, SLC9C1 contains a voltage-sensing domain (VSD) and a cyclic nucleotide-binding domain (CNBD) that regulate its activity, as revealed by studies in sea urchins (15–17).

Knockout (KO) studies have shown that SLC9C1 deficiency causes immotile spermatozoa and male infertility in mice, which can be rescued by cell-permeable cAMP analogs or photoactivated adenylyl cyclase, underscoring the interplay between SLC9C1 and cAMP production via sAC (14, 18). Several transcript variants are present in sAC, including full-length (sAC_fl_) and truncated (sAC_t_) forms, both are expressed in male germ cells. In *Slc9c1* KO mouse models, the amount of sAC_fl_ but not sAC_t_ decreased in mature spermatozoa (19), indicating that sAC_fl_ is associated with SLC9C1 for the cAMP production. In addition to mice, clinical studies link pathogenic variants in *SLC9C1* or *ADCY10* to asthenozoospermia and male infertility in humans (5, 20). The existence of both *SLC9C1* and *ADCY10* has been confirmed across Metazoa, suggesting that they are conserved functional partners (21, 22).

In this study, we identified TMEM217, a transmembrane protein expressed predominantly in testes, as a key factor coevolving with SLC9C1. Our data reveal that TMEM217 interacts with the VSD of SLC9C1, playing a pivotal role in forming functional spermatozoa. Moreover, we found that the presence of TMEM217 is essential for sustaining the amount of sAC_fl_ in spermatozoa through SLC9C1, which facilitates cAMP production.

## Results

### *SLC9C1* Coevolves with *SLC9C2* and *TMEM217*.

**Fig. 1.**
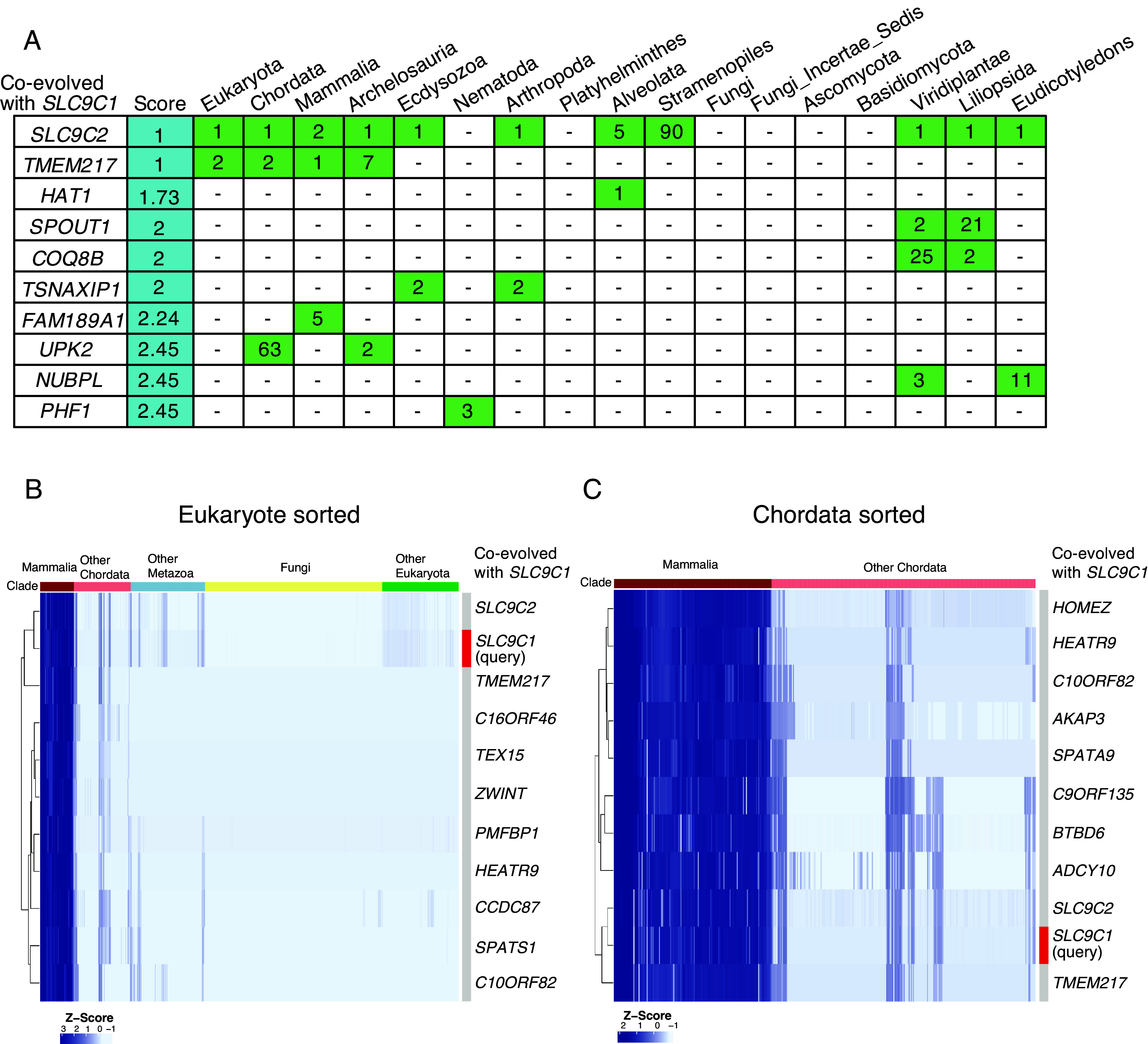
Coevolving genes with *SLC9C1*. (*A*) Coevolving genes with *SLC9C1* were identified using the CladeOScope web platform. The 10 genes with the lowest CladeOScope scores, indicating strong coevolution across 17 clades, are shown. Each cell represents the ranking of genes in each clade based on the Pearson correlation with the phylogenetic profile of *SLC9C1*. Ranks greater than 100 are omitted and presented as a blank cell. (*B* and *C*) Heatmap showing the phylogenetic profiles of the top 10 coevolving genes within Eukaryotes (*B*) and Chordata (*C*). Conservation levels are color-coded, with darker blue indicating higher conservation. Genes are clustered according to their conservation patterns, as shown in the dendrogram on the *Left*. Each row corresponds to a gene, and each column to a species.

To understand functional partners of SLC9C1, we examined its evolutionary relationships with other genes. To identify coevolving genes with *SLC9C1*, we used the CladeOScope, which applies phylogenetic profiling to detect coevolutionary relationships across and within specific clades (23). *SLC9C2*, a homolog of *SLC9C1*, was ranked as the highest coevolving gene with *SLC9C1*, based on the CladeOScope score (Fig. 1*A*). In addition, *TMEM217*, a gene with an uncharacterized function, was identified as the top coevolving gene with *SLC9C1*. *TMEM217* was ranked highly in Eukaryota, Chordata, Mammalia, and Archelosauria (Fig. 1*A*).

We next assessed the conservation of top 10 coevolving genes with *SLC9C1* across Eukaryota using the heatmap of phylogenetic profiles (Fig. 1*B*). *TMEM217* was highly conserved in mammals and also conserved in some reptiles and fish, suggesting an important role that may have emerged later in vertebrate evolution (NCBI Gene, Gene ID: 221468) (Fig. 1*B* and *SI Appendix,* Fig. S1). *SLC9C1*, *SLC9C2*, and *TMEM217* exhibited overlapping conservation patterns (Fig. 1 *B* and *C*). Importantly, analysis within Chordata also identified *ADCY10* as a coevolving gene with *SLC9C1*, consistent with previous reports demonstrating their functional interaction in sperm physiology (19) and supporting their evolutionary relationship (21, 22) (Fig. 1*C*). These data suggest that *SLC9C2* and *TMEM217* coevolve with *SLC9C1*, and their encoding proteins are potentially functional partners of SLC9C1.

### TMEM217 Is Localized in the Principal Piece of Spermatozoa.

While both *SLC9C2* and *TMEM217* were identified as top coevolving partners of *SLC9C1*, *Slc9c2* was reported to be a pseudogene in mice, limiting its functional studies using mouse models (13). In contrast, *TMEM217* is functionally uncharacterized, yet exhibits strong coevolutionary signals with *SLC9C1*, and is conserved in mice (RefSeq: NP_001156373.1, NP_001156374.1). Therefore, we analyzed *TMEM217* to explore its functions.

**Fig. 2.**
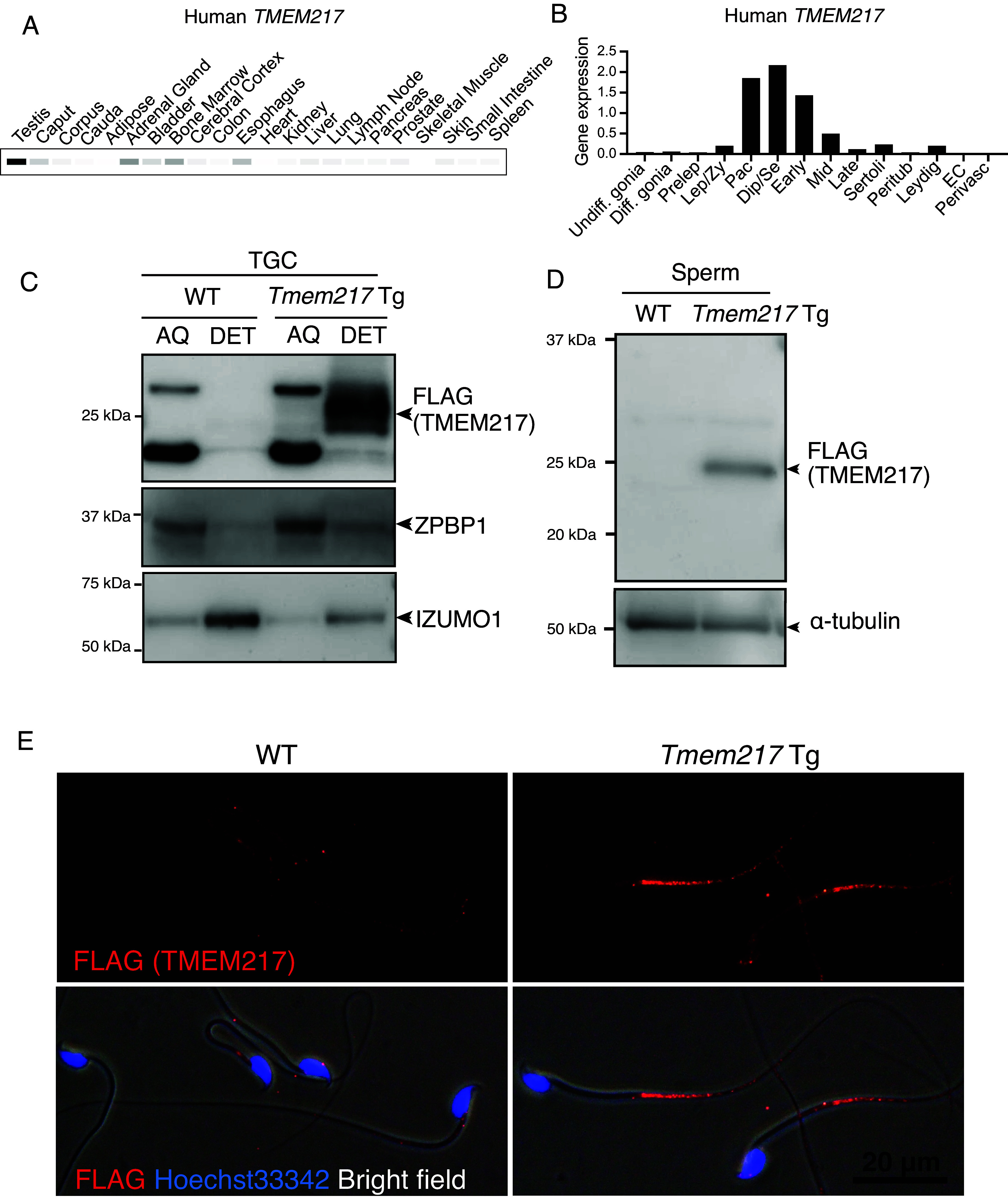
TMEM217 is expressed predominantly in male germ cells and localized at the principal piece of spermatozoa. (*A*) Expression pattern of *TMEM217* in human tissues. Band intensities are based on the average transcripts per million (TPM). White = 0 TPM, Black ≥ 30 TPM. (*B*) Expression profile of human *TMEM217* in testicular cells using previously published datasets (29). Undiff. gonia: Undifferentiated spermatogonia, Diff. gonia: differentiating spermatogonia, Prelep: preleptotene spermatocytes, Lep/Zy: leptotene and zygotene spermatocytes, Pac: pachytene spermatocytes, Dip/Se: diplotene and secondary spermatocytes, Early: early round spermatids, Mid: mid round spermatids, Late: late round spermatids, Peritub: peritubular myoid cells, EC: Endothelial cells, and Perivasc: perivascular cells. (*C*) Phase separation of Triton X-114 extracts of TGCs of WT mice and *Tmem217* Tg mice. TMEM217-3xFLAG in Tg mice was enriched in the detergent-enriched (DET) phase. ZPBP1 is a marker for the aqueous (AQ) fraction, whereas IZUMO1 is a marker for the DET fraction. (*D*) Immunoblot analyses of mature spermatozoa collected from the epididymis. α-tubulin was used as a loading control. (*E*) Subcellular localization of TMEM217-3xFLAG was analyzed in mature spermatozoa. Nuclei were stained with Hoechst 33342 (blue). Nonspecific signals of FLAG (TMEM217) were detected in the midpieces of both WT and *Tmem217* Tg spermatozoa.

A gene encoding TMEM217 is located on chromosome 6 in humans and chromosome 17 in mice. Mammalian Reproductive Genetics Database (24–27) shows that human and mouse *Tmem217* are both expressed predominantly in testes (Fig. 2*A* and *SI Appendix,* Fig. S2*A*). Predominant expression of *Tmem217* in mouse testes was confirmed by RT-PCR (*SI Appendix,* Fig. S2*B*). Further, single-cell RNA-sequencing datasets of testes show that human and mouse *Tmem217* start to express in pachytene spermatocytes (Fig. 2*B* and *SI Appendix,* Fig. S2*C*). Consistently, RT-PCR analyses using mouse testes at different ages showed that *Tmem217* expression increased gradually from postnatal day 18 (*SI Appendix,* Fig. S2*D*), which corresponds to the appearance of mid to late spermatocytes during the first wave of spermatogenesis (28).

To clarify the localization of TMEM217 in mouse testicular cells, we generated several anti-TMEM217 antibodies, but none detected endogenous TMEM217 with immunostaining or immunoblot analyses. To compensate, we generated transgenic (Tg) mice expressing C-terminally 3xFLAG-tagged *Tmem217* under the testis-specific *Clgn* promoter (*SI Appendix,* Fig. S2*E*). The timing of *Clgn* expression is similar to that of *Tmem217* during mouse spermatogenesis (*SI Appendix,* Fig. S2*F*). Using testicular germ cells (TGCs) of Tg mice, we found that TMEM217-3xFLAG was enriched in the Triton X-114 detergent-phase fraction that contains membrane-associated proteins (Fig. 2*C*). Further, TMEM217*-*3xFLAG was detected in the principal piece of cauda epididymal sperm flagella, similar to SLC9C1 (14) (Fig. 2 *D* and *E*).

### KO of *Tmem217* Abolishes Male Fertility due to Sperm Immotility.

**Fig. 3.**
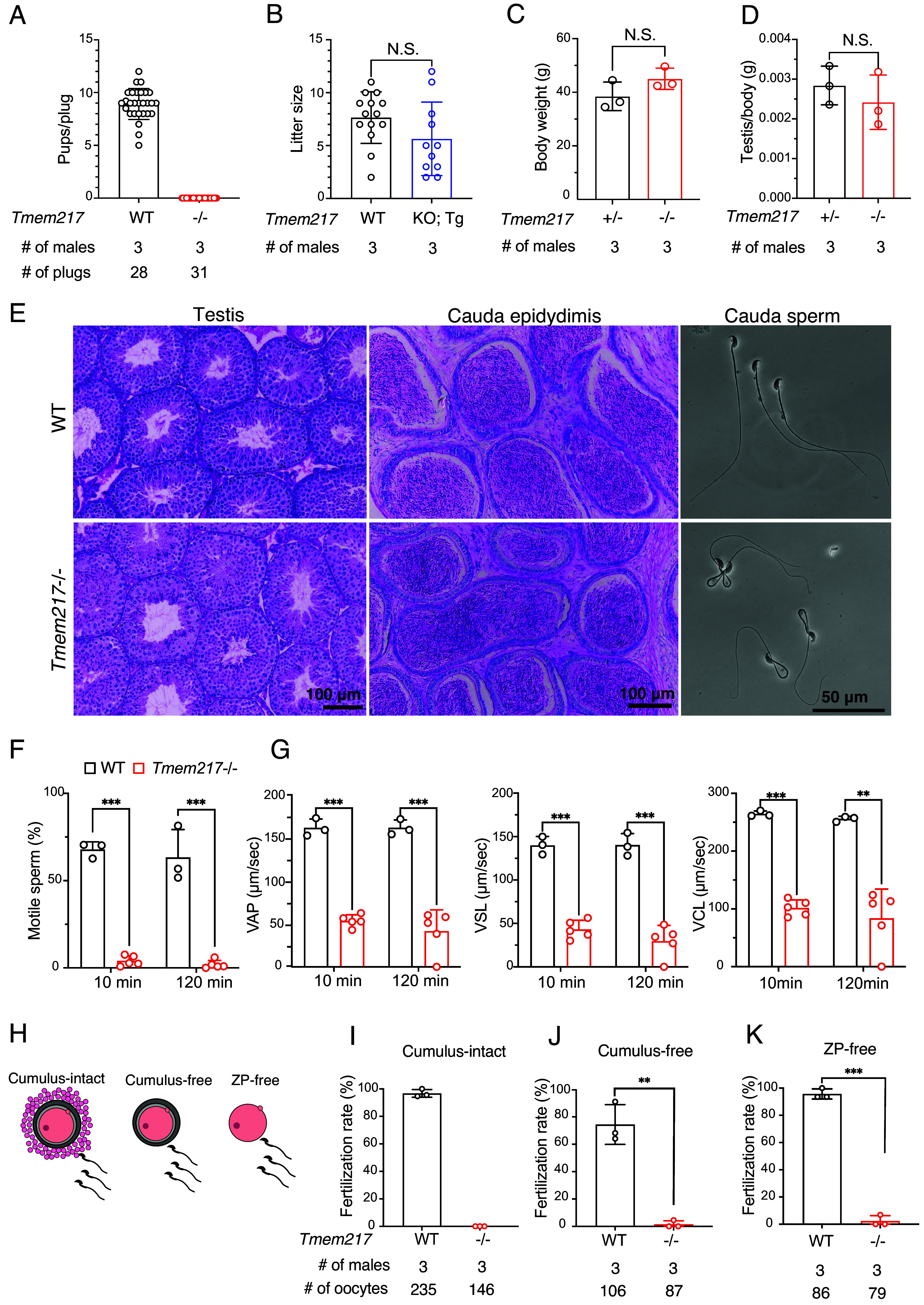
*Tmem217* KO abolishes male fertility due to reduced sperm motility. (*A*) Mating tests of WT and KO male mice were performed. The number of pups per plug is shown. (*B*) Mating tests of WT and Tg male mice were performed. Litter size is shown. (*C*) Body weight of control and KO male mice. N.S.: not significant. (*D*) Testis weight normalized to body weight in control and KO male mice. (*E*) Hematoxylin and periodic acid–Schiff (HePAS) staining of testis and cauda epididymis sections, and phase contrast images of mature spermatozoa. (*F* and *G*) The percentage of motile spermatozoa (*F*) and velocity parameters (*G*), such as VAP (average path velocity), VSL (straight-line velocity), and VCL (curvilinear velocity), were measured after 10-min and 120-min incubation in capacitation medium (n = 3 for WT males, n = 5 for KO males). VAP, VSL, and VCL values were measured only with motile spermatozoa. (*H*) Cumulus-intact, cumulus-free, and zona pellucida-free (ZP-free) oocytes were used for in vitro fertilization (IVF). (*I*–*K*) IVF results. Cumulus-intact (*I*), cumulus-free (*J*), and ZP-free (*K*) oocytes were used. The formation of two pronuclei (2PN) was taken as an indication of successful fertilization. Error bars represent SD. *P* values were determined by a two-tailed unpaired Student’s *t* test. *P* < 0.01 (**), and *P* < 0.001 (***).

To investigate the roles of TMEM217 in male fertility, we generated *Tmem217* KO mice. Two guide RNAs (gRNAs) were designed to target the upstream and downstream regions of the coding sequence of exon 3, respectively, and introduced into 68 zygotes via electroporation. Sixty embryos at the two-cell stage were obtained, and 40 embryos were transferred into the oviducts of pseudopregnant females. Three out of five F0 pups carried large deletions. By performing subsequent mating of F0 mice, we generated *Tmem217* KO mice with a 1,071 bp deletion (*SI Appendix,* Fig. S3 *A* and *B*). No obvious abnormalities were found in development or behavior in *Tmem217* KO mice. *Tmem217* KO males were then subjected to mating tests, where each male was housed with three adult females for 2 mo. While over 30 vaginal plugs were observed, no offspring were obtained from *Tmem217* KO males (Fig. 3*A*), indicating that *Tmem217* is essential for male fertility. We confirmed that male fertility was restored in *Tmem217* KO mice carrying a transgene encoding TMEM217 (Fig. 3*B* and *SI Appendix*, Fig. S2*E*), demonstrating that infertility was not due to off-target effects.

To understand the cause of male infertility, we first analyzed testes, but no abnormalities were found in weights or sections of *Tmem217* KO testes (Fig. 3 *C*–*E*). Furthermore, no abnormalities were found in sections of *Tmem217* KO cauda epididymis (Fig. 3*E*). We then analyzed mature spermatozoa obtained from the cauda epididymis and found a hairpin-like bending in *Tmem217* KO sperm flagella in isotonic medium (TYH medium, ~310 mOsm) (Fig. 3*E*). To evaluate whether the hairpin-like bending is associated with defective osmoregulation, we examined sperm morphology in varying osmolarity conditions adjusted with NaCl (30, 31). We first checked sperm viability under each condition and found no differences between WT and *Tmem217* KO mice in all conditions (*SI Appendix,* Fig. S3 *C* and *D*). We then observed sperm morphology and found that *Tmem217* KO spermatozoa showed straight flagella in a hypertonic medium that mimics the cauda epididymal environment (440 mOsm; *SI Appendix,* Fig. S3 *C* and *E*). Conversely, under a hypotonic condition (150 mOsm), even WT spermatozoa exhibited a higher percentage of flagellum bending, which is comparable to *Tmem217* KO spermatozoa. These findings suggest that *Tmem217* KO spermatozoa have defects in osmoregulation (31), which may lead to the hairpin-like bending of flagella in isotonic medium.

Because sperm flagellum morphology is associated with motility, we analyzed sperm motility using a computer-assisted sperm analysis system. The majority of *Tmem217* KO spermatozoa were immotile, and the small fraction of spermatozoa that displayed motility exhibited VAP, VSL, and VCL values around 30% of those observed in WT spermatozoa at both 10 and 120 min incubations (Fig. 3 *F* and *G* and Movies S1–S4). We also performed in vitro fertilization and revealed that *Tmem217* KO spermatozoa failed to fertilize cumulus-intact oocytes (Fig. 3 *H* and *I*). The fertilizing ability was not rescued by removing cumulus cells or both cumulus cells and the zona pellucida (Fig. 3 *H*, *J*, and *K*), confirming the impaired fertilizing ability of *Tmem217* KO spermatozoa. Altogether, these results indicate that *Tmem217* KO males are infertile due to abnormal sperm morphology (bending flagella) and impaired sperm motility.

### TMEM217 Interacts with SLC9C1 during Spermatogenesis.

**Fig. 4.**
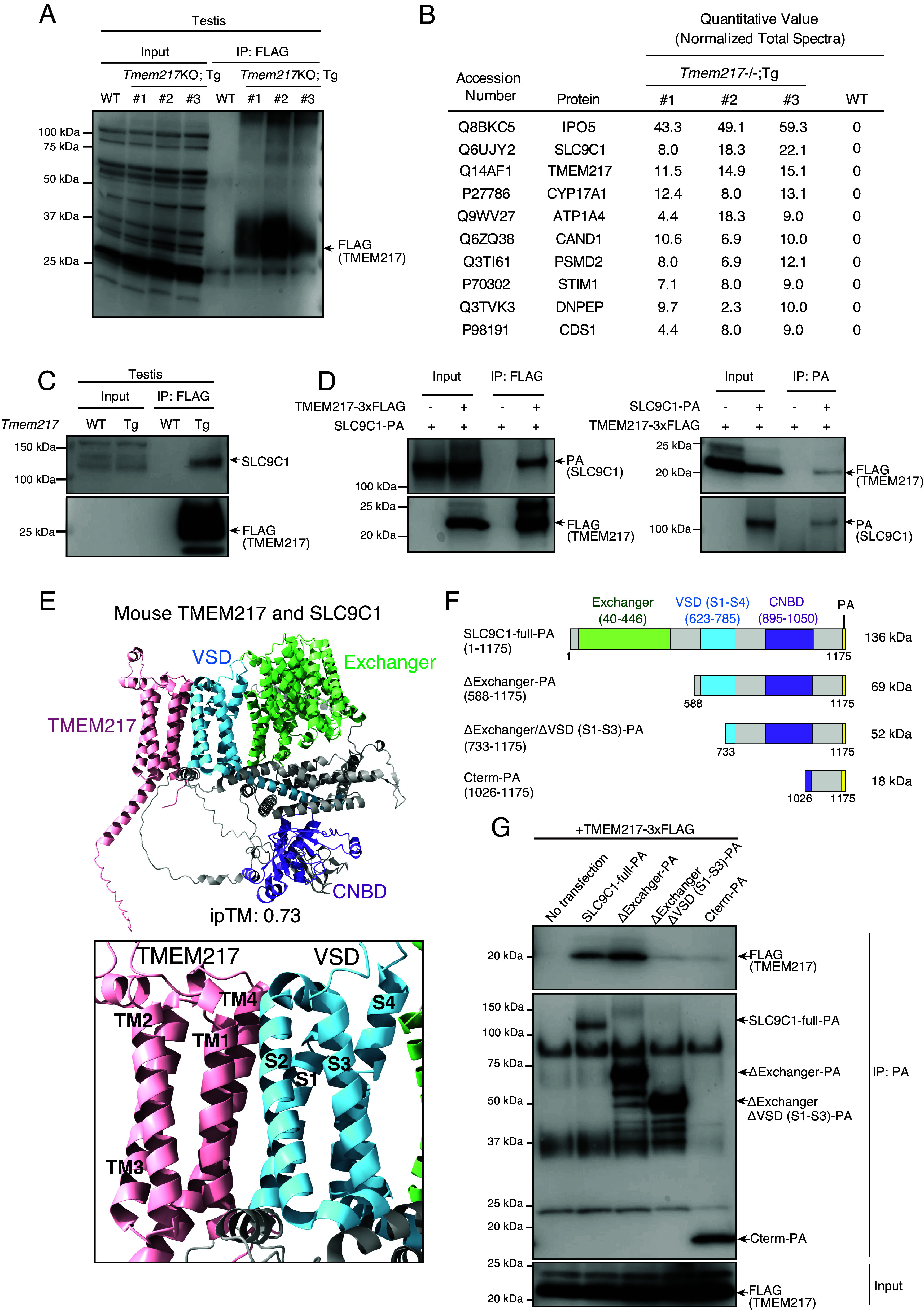
TMEM217 interacts with SLC9C1 in mouse testes. (*A*) Immunoblot analysis detecting TMEM217-3xFLAG after FLAG IP of testis samples from WT and three independent *Tmem217* KO; Tg mice (#1 – #3). (*B*) Top 10 proteins ranked by quantitative values identified via LC–MS analysis following FLAG IP of WT and *Tmem217* KO; Tg testes (#1 – #3). (*C*) Immunoblot analysis detecting SLC9C1 and TMEM217-3xFLAG after FLAG IP of testis samples. (*D*) Co-IP analysis using anti-FLAG (*Left*) and anti-PA (*Right*) antibodies on HEK293T cell lysates expressing mouse TMEM217-3xFLAG and mouse SLC9C1-PA. (*E*, *Top*) AlphaFold3 3D structural prediction of the mouse TMEM217–SLC9C1 complex. SLC9C1 possesses Na^+^/H^+^ exchanger domain (Exchanger), VSD, and CNBD. The interface predicted template modeling (ipTM) score of the complex is shown. (*Bottom*) A close-up view of the TMEM217 and VSD of SLC9C1 interface. Transmembrane helices TM1–TM4 of TMEM217 and S1–S4 of the VSD are labeled. (*F*) Schematic representation of SLC9C1 domains analyzed in co-IP experiments shown in (*G*). (*G*) Identification of the TMEM217-binding region of SLC9C1 with co-IP. Truncated SLC9C1-PA constructs shown in (*F*) were coexpressed with TMEM217-3xFLAG, followed by co-IP using PA antibody. FLAG and PA antibodies were used for immunoblotting.

TMEM217 is required for sperm osmoregulation and motility; however, it does not have any known functional domains. To understand the molecular functions of TMEM217, we performed an interactome analysis using *Tmem217-3xFLAG* Tg mice. TMEM217*-*3xFLAG was immunoprecipitated using an anti-FLAG antibody from testis lysates, and mass spectrometry was performed to identify interacting proteins (Fig. 4 *A* and *B* and Dataset S1). We conducted gene ontology (GO) enrichment analyses on identified proteins. GO terms such as “Establishment of protein localization to organelle,” “Intraciliary retrograde transport,” and “Protein N-linked glycosylation” were enriched, suggesting that interacting proteins may be involved in TMEM217 localization to the sperm flagellum and its posttranslational modification (*SI Appendix,* Fig. S4 *A* and *B* and Dataset S2). Among these interactors, proteins identified in *Tmem217*-*3xFLAG* Tg testes with high quantitative values but not in WT testes are listed in Fig. 4*B*. Intriguingly, we found SLC9C1 in the list, consistent with the coevolutionary analysis (Fig. 1). We confirmed that TMEM217 interacts with SLC9C1 in testes with immunoblot analysis following immunoprecipitation (IP) (Fig. 4*C*).

To further investigate the interaction between TMEM217 and SLC9C1, mouse *Tmem217-3xFLAG* was coexpressed with full-length mouse *Slc9c1*-*PA* in HEK293T cells, and their protein–protein interactions were analyzed by IP. The result confirmed that mouse TMEM217 interacts with mouse full-length SLC9C1 (Fig. 4*D*). Furthermore, co-IP experiments using human *TMEM217-PA* and *SLC9C1-3xFLAG* constructs demonstrated their interaction as well (*SI Appendix,* Fig. S4*C*).

In addition to the exchanger domain commonly found in SLC9 family proteins, SLC9C1 possesses VSD (four transmembrane domains S1–S4), and CNBD (14–17) (Fig. 4*E*). Structural predictions by AlphaFold3 suggest that mouse and human TMEM217 interact with SLC9C1 through the VSD, with the S1, S2 segments in close proximity (Fig. 4*F* and *SI Appendix,* Fig. S4*D*). To confirm the actual interacting domain, various *Slc9c1* constructs were generated, including full-length *Slc9c1*-*PA*, ΔExchanger-PA (lacking the exchanger domain), ΔExchanger/ΔVSD (S1–S3)-PA (lacking the exchanger domain and the VSD region, including S1 to S3 and the N-terminal part of S4), and Cterm-PA (containing the C-terminal part of CNBD to the C-terminus of SLC9C1) (Fig. 4*F*). These constructs were coexpressed with mouse *Tmem217*-*3xFLAG* in HEK293T cells. IP revealed that TMEM217-3xFLAG was pulled down efficiently by full-length SLC9C1-PA and ΔExchanger-PA. In contrast, the signals were diminished when immunoprecipitated by ΔExchanger/ΔVSD (S1–S3)-PA or Cterm-PA (Fig. 4 *F* and *G*). These results indicate that TMEM217 interacts with SLC9C1 through the VSD.

Structural prediction by AlphaFold3 indicates that the interaction between TMEM217 and SLC9C1 through the VSD is conserved across diverse species, including lungfish, turtles, and marsupials, suggesting that this interaction emerged before the divergence of mammals and has been maintained in distinct phylogenetic lineages (*SI Appendix,* Fig. S5 *A* and *B*).

### KO of *Tmem217* Depletes SLC9C1 and Full-Length sAC in Spermatozoa.

**Fig. 5.**
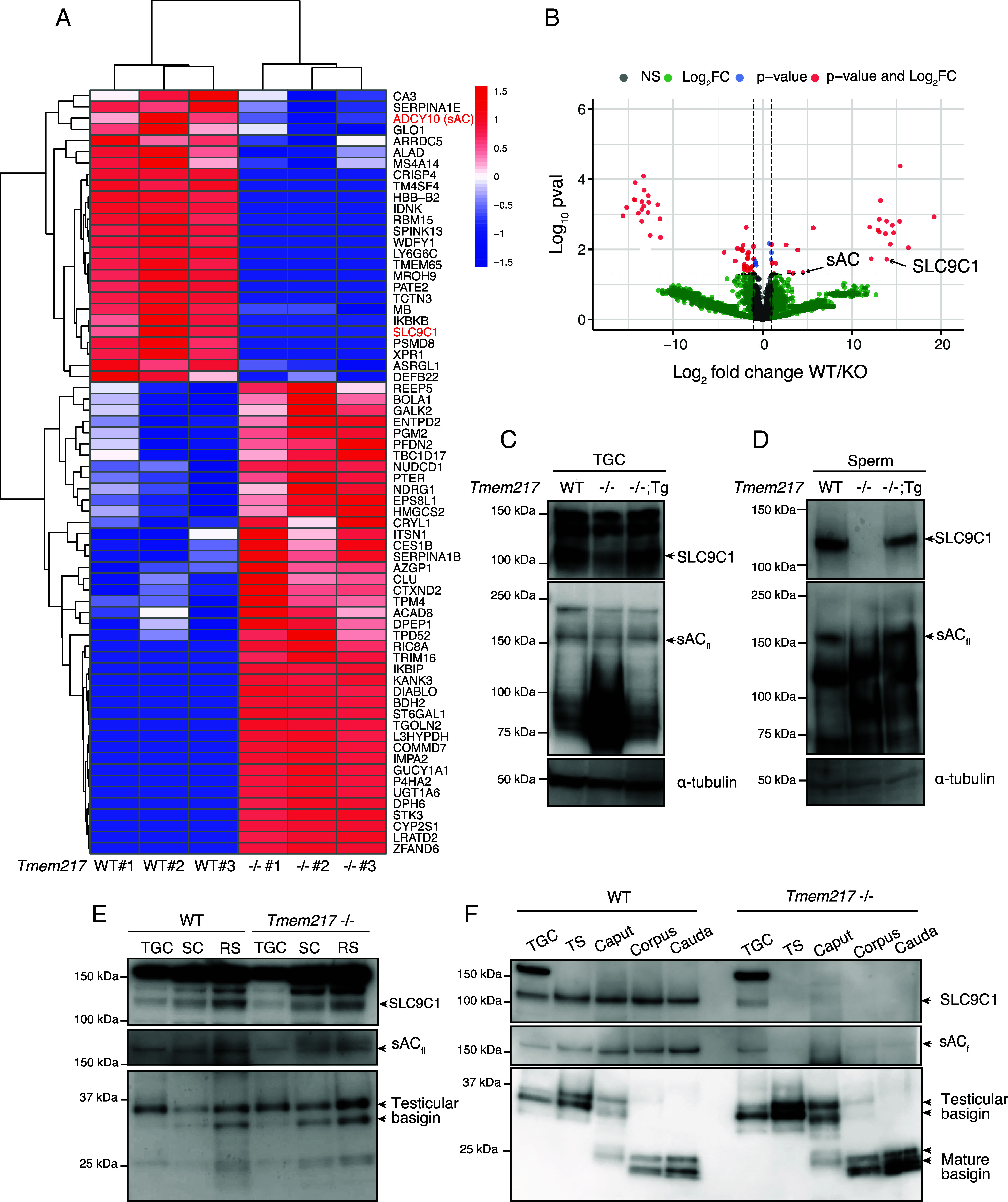
*Tmem217* KO results in the loss of SLC9C1 in mature spermatozoa. (*A* and *B*) NanoLC-MS/MS analysis of mature spermatozoa was performed (n = 3 males for each genotype). Heatmap showing significantly upregulated and downregulated proteins (*A*). Proteins were identified using a two-tailed unpaired Welch’s *t* test (*P* < 0.05) (log2 fold change > 1 or log2 fold change < −1). Volcano plot illustrating differential protein expression (*B*). Red points denote proteins with significant changes (*P* < 0.05) and log2 fold change values exceeding 1 or below −1. (*C*) Immunoblot analysis of SLC9C1 and sAC protein levels in TGC from WT, *Tmem217* KO, and *Tmem217* KO; Tg mice. α-tubulin was used as a loading control. sAC_fl_: full-length sAC. (*D*) Immunoblot analysis of SLC9C1 and sAC protein levels in mature spermatozoa from WT, *Tmem217* KO, and *Tmem217* KO; Tg cauda epididymis. α-tubulin was used as a loading control. (*E*) Immunoblot analysis of SLC9C1 and sAC protein levels in TGC, spermatocyte (SC), and round spermatid (RS). Basigin was used as a loading control. (*F*) Immunoblot analysis of SLC9C1 and sAC protein levels in TGC, testicular spermatozoa (TS), and spermatozoa isolated from different regions of the epididymis (caput, corpus, and cauda). Basigin was used as a loading control.

To understand the effects of TMEM217 deletion on the sperm proteome, we performed mass spectrometry on mature spermatozoa collected from the cauda epididymis. Our primary aim was to assess whether the amount of SLC9C1 was reduced in *Tmem217* KO mature spermatozoa; however, SLC9C1 peptides were undetectable in the data-dependent acquisition shotgun proteomic analysis, even in WT spermatozoa, suggesting that SLC9C1 may be present at very low levels. To overcome this limitation, we utilized data-independent acquisition analysis after fractionation of the sperm lysate proteins with a trifluoroacetic acid gradient. Using this analysis, we detected a total of ~7,013 proteins (Dataset S3). Intriguingly, SLC9C1 peptides were detected in all three WT samples, but not in *Tmem217* KO mature spermatozoa (Fig. 5 *A* and *B* and Dataset S3). We further analyzed the amount of SLC9C1 with immunoblotting. In TGCs, the amount of SLC9C1 decreased in *Tmem217* KO mice compared to WT mice (Fig. 5*C*). Moreover, SLC9C1 was not detected in *Tmem217* KO mature spermatozoa. At the same time, it was present in spermatozoa of *Tmem217* KO mice with *Tmem217-3xFLAG* transgene (Fig. 5*D*), indicating that TMEM217 is essential for the presence of SLC9C1 in mature spermatozoa.

Previous studies have shown that *Slc9c1* KO mice exhibit a significant reduction of ~180 kDa sAC_fl_ in mature spermatozoa (19). Our mass spectrometry analyses confirmed that total sAC was significantly reduced in mature spermatozoa of *Tmem217* KO mice (Fig. 5 *A* and *B* and Dataset S3). Like *Slc9c1* KO mice, immunoblot analyses showed that the amount of sAC_fl_ was reduced in mature *Tmem217* KO spermatozoa (Fig. 5 *C* and *D*). These results indicate that the loss of SLC9C1 in *Tmem217* KO spermatozoa decreased the amount of sAC_fl_.

To further investigate the loss of SLC9C1 and sAC_fl_ in *Tmem217* KO mice, we attempted immunostaining of SLC9C1 in testes, but the anti-SLC9C1 antibody failed to produce a detectable signal in immunofluorescence. Instead, we isolated spermatocytes, round spermatids (Fig. 5*E* and *SI Appendix,* Fig. S6 *A* and *B*), testicular spermatozoa, and spermatozoa from the caput, corpus, and cauda epididymis (Fig. 5*F* and *SI Appendix,* Fig. S6*C*). Immunoblot analyses revealed that the protein levels of SLC9C1 and sAC_fl_ in spermatocytes were not reduced by the absence of TMEM217. However, in later stages, their levels were lower in *Tmem217* KO mice compared to WT, and were lost in testicular spermatozoa (Fig. 5 *E* and *F*), suggesting that SLC9C1 and sAC_fl_ are lost during spermiogenesis.

### SLC9C1 Can Localize to the Plasma Membrane without TMEM217.

We further analyzed the relationship between TMEM217 and SLC9C1 using HEK293T cells. We expressed both *Tmem217* and *Slc9c1*, but coexpression did not affect the amount of SLC9C1 (*SI Appendix,* Fig. S7*A*). To rule out the possibility that endogenous TMEM217 is sufficient to sustain the amount of SLC9C1, we deleted the *TMEM217* gene in HEK293T cell lines with the CRISPR/Cas9 system using two gRNAs (*SI Appendix,* Fig. S7 *B* and *C*). The absence of TMEM217 in HEK293T cells did not alter the SLC9C1 protein level (*SI Appendix,* Fig. S7*D*). Furthermore, TMEM217 deficiency did not disrupt the plasma membrane localization of SLC9C1 (*SI Appendix,* Fig. S7*E*). These results indicate that SLC9C1 can localize to the plasma membrane without TMEM217. Considering that TMEM217 and SLC9C1 are both localized in the principal piece of mature spermatozoa (Fig. 2*E*) (14) and lost during spermiogenesis, flagellum localization of the TMEM217-SLC9C1 complex, but not plasma membrane localization, may be disrupted in *Tmem217* KO mice, which leads to the loss of SLC9C1 and sAC_fl_ in spermatozoa.

### *Tmem217* KO Spermatozoa Exhibit a Reduced Amount of cAMP.

**Fig. 6.**
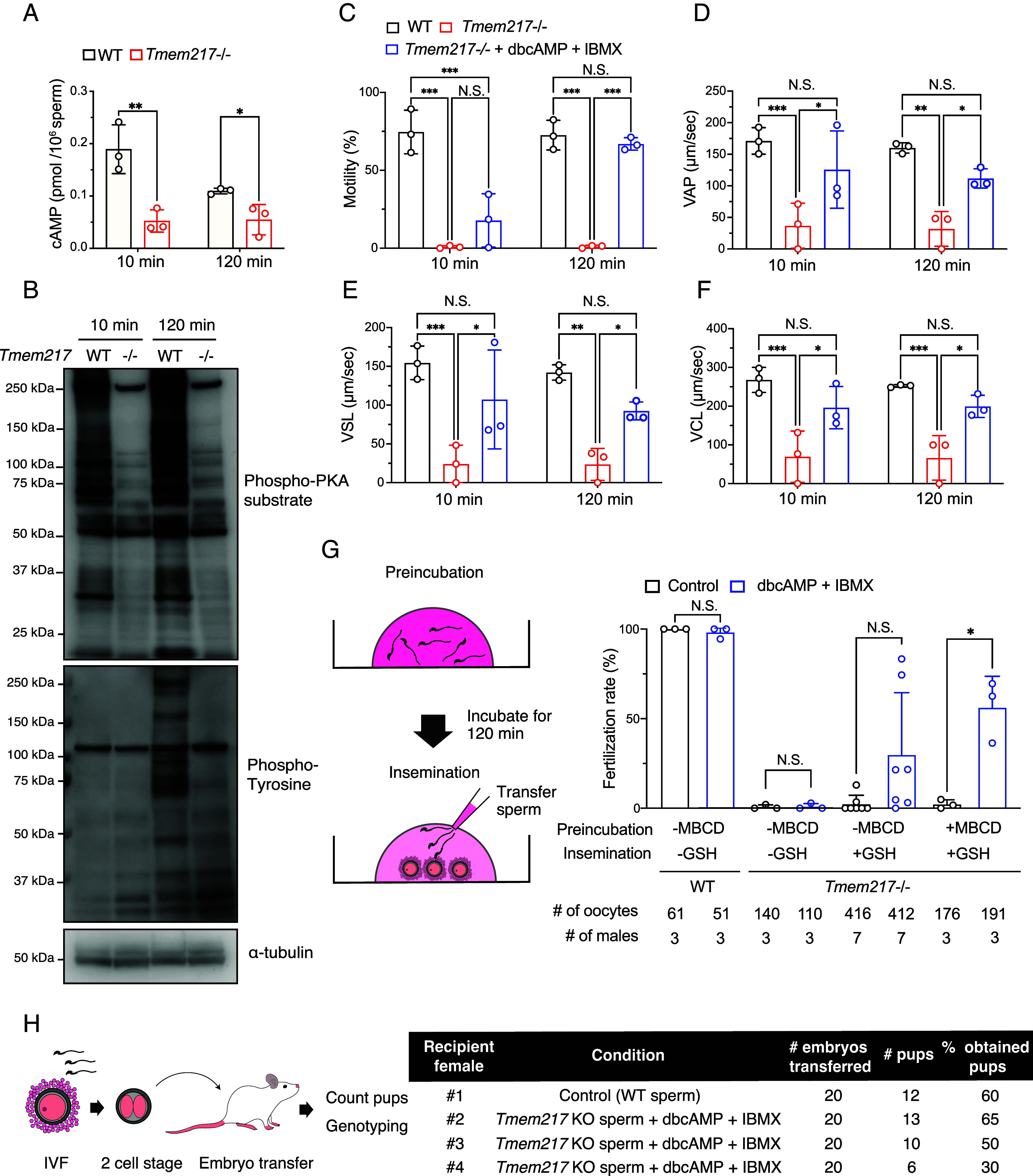
Rescue of sperm motility and fertilizing ability in *Tmem217* KO mice. (*A*) Quantification of intracellular cAMP levels in mature spermatozoa from WT and *Tmem217* KO cauda epididymis after 10- and 120-min incubation in capacitation medium (n = 3 males for each data point). (*B*) Immunoblot analysis of phospho-PKA substrate and phospho-tyrosine levels in mature spermatozoa from WT and *Tmem217* KO cauda epididymis after 10- and 120-min incubation in capacitation medium. α-tubulin was used as a loading control. (*C*) Percentage of motile spermatozoa after 10- and 120-min incubation in capacitation medium for WT, *Tmem217* KO (without dbcAMP and IBMX), and *Tmem217* KO (with dbcAMP and IBMX) groups (n = 3 males for each data). (*D*–*F*) Velocity parameters of sperm motility, including average path velocity (VAP) (*D*), straight-line velocity (VSL) (*E*), and curvilinear velocity (VCL) (*F*), after 10- and 120-min incubation in capacitation medium (n = 3 males for each data). (*G*, *Left*) Illustration of the IVF experiment. Spermatozoa were incubated in preincubation medium with or without MBCD for 120 min. Subsequently, spermatozoa and oocytes were incubated in insemination medium with or without GSH. (*Right*) IVF outcomes using spermatozoa from WT, *Tmem217* KO (without dbcAMP and IBMX), and *Tmem217* KO (with dbcAMP and IBMX). The formation of 2PN was taken as an indication of successful fertilization. (*H*, *Left*) *Tmem217* KO spermatozoa incubated with dbcAMP and IBMX were used for IVF, with WT spermatozoa as the control. After fertilization, embryos developed to the two-cell stage and were transferred into the ampulla of pseudopregnant female mice. (*Right*) Total number and percentage of pups obtained from IVF using dbcAMP and IBMX. *P* values for (*A* and *G*) were determined using a two-tailed unpaired Student’s *t* test. *P* values for (*C*–*F*) were determined using one-way ANOVA followed by Tukey’s multiple comparisons test. Error bars represent SD. *P* < 0.05 (*), *P* < 0.01 (**), and *P* < 0.001 (***).

Spermatozoa from *Tmem217* KO mice showed severe motility defect (Fig. 3 *F* and *G*), similar to *Slc9c1* KO mice (14). Sperm motility of *Slc9c1* KO mice has been reported to recover after incubation with cAMP analogs (14), suggesting that impaired sperm motility is caused by the decreased amount of sAC_fl_ and subsequent abnormal cAMP production (19). We then analyzed cAMP in mature spermatozoa at the time of incubation in capacitation medium for 10 min, when cAMP levels were reported to be at their maximum (32), and 120 min, and found that the amounts of cAMP were significantly reduced in *Tmem217* KO spermatozoa at both periods (Fig. 6*A*). It has been reported that PKA signaling pathways activated by cAMP are necessary for vigorous sperm motility and capacitation (33). Consistently, we found that phosphorylation levels of PKA substrates were significantly downregulated in *Tmem217* KO spermatozoa at both 10- and 120-min incubations in capacitation medium (Fig. 6*B*). Furthermore, because PKA activation triggers protein tyrosine phosphorylation in mature spermatozoa (34), we analyzed phospho-tyrosine levels. While tyrosine phosphorylation increased after 120-min incubation in WT spermatozoa, impaired tyrosine phosphorylation was found in *Tmem217* KO spermatozoa (Fig. 6*B*). These results indicate that the absence of TMEM217 causes reduced cAMP production and impaired downstream signaling pathways.

### cAMP Analog Rescues Motility and Fertility in *Tmem217* KO Spermatozoa.

To understand whether impaired sperm motility of *Tmem217* KO mice is caused by insufficient cAMP production, we incubated spermatozoa with dibutyryl-cAMP (dbcAMP), a cell-permeable cAMP analog, and IBMX, a phosphodiesterase inhibitor (4, 14, 18). *Tmem217* KO spermatozoa incubated in capacitation medium supplemented with dbcAMP and IBMX exhibited limited motility recovery at 10-min incubation (Fig. 6*C* and Movie S5) and a substantial recovery was observed at 120-min incubation (Fig. 6*C* and Movie S6). Moreover, velocity parameters such as VAP, VSL, and VCL were improved at both 10- and 120-min incubations (Fig. 6 *D*–*F*).

To reveal whether the fertilizing ability of *Tmem217* KO spermatozoa was also rescued, we performed IVF after preincubation of spermatozoa with dbcAMP and IBMX. When we used common capacitation medium (TYH) for both sperm preincubation and insemination, the fertilizing ability of *Tmem217* KO spermatozoa was not rescued by dbcAMP and IBMX (Fig. 6*G*). A previous report has shown that cAMP analogs rescue the fertilizing ability of *Slc9c1* KO spermatozoa only when oocytes without zona pellucida are used (14). In addition, the fertilizing ability of sAC (*Adcy10*) null spermatozoa was not rescued with dbcAMP (4). In contrast, cAMP production induced by the light stimulation of photoactivated adenylyl cyclase bPAC transgene rescued the fertilizing ability of *Slc9c1* KO spermatozoa using oocytes with zona pellucida (18). These data suggest that an increase in the intracellular cAMP level has the potential to compensate for deficient cAMP production due to a decreased amount of sAC, although the addition of cAMP analogs is not as effective. This could be attributed to the insufficient amount of cAMP analogs reaching inside the cells or the inability of cAMP analogs to activate downstream signaling pathways in a manner comparable to WT endogenous cAMP (18). Therefore, we conducted IVF utilizing a sperm preincubation medium comprising methyl-β-cyclodextrin (MBCD) (FERTIUP), which was shown to enhance cholesterol efflux and plasma membrane fluidity and facilitate downstream signal transduction (35–37). Furthermore, we used an insemination medium (CARD MEDIUM) containing reduced glutathione (GSH) that facilitates IVF with oocytes with zona pellucida (38, 39). Notably, the use of MBCD and GSH alone did not rescue the fertilizing ability of *Tmem217* KO spermatozoa (Fig. 6*G*). However, the addition of dbcAMP and IBMX to these media did rescue the fertilizing ability of *Tmem217* KO spermatozoa (Fig. 6*G*). Furthermore, fertilized oocytes developed to the two-cell stage, and 20 two-cell embryos in each trial were transferred to pseudopregnant females. As a result, 28 *Tmem217* heterozygous pups were born (Fig. 6*H* and *SI Appendix,* Fig. S8). These results indicate that the infertility of *Tmem217* KO mice is primarily caused by deficient cAMP production, which is likely due to the absence of SLC9C1 and sAC_fl_.

## Discussion

SLC9C1 is essential for sperm motility through its role in integrating cAMP signaling (13, 14, 17–19, 40). Here, we demonstrate that *TMEM217* is a coevolving gene with *SLC9C1* and vital for sperm motility and male fertility in mice. *Tmem217* KO spermatozoa showed the loss of SLC9C1, which disrupts sAC_fl_ levels and cAMP production, subsequently affecting signal transduction necessary for sperm motility. Importantly, supplementation with cell-permeable cAMP rescued the motility and fertilizing ability of *Tmem217* KO spermatozoa, highlighting the importance of TMEM217 in regulating SLC9C1-mediated cAMP production.

From an evolutionary perspective, SLC9C1 and sAC orthologs are present in invertebrates, including cnidarians and echinoderms, indicating the conservation of regulatory mechanisms in pH_i_ and cAMP-dependent sperm motility in these invertebrate species (21, 22). In contrast, TMEM217 is highly conserved in mammals, only sporadically conserved in reptiles and fish, and absent in birds and invertebrates. This conservation pattern suggests that the mechanism for localizing SLC9C1 in spermatozoa evolved alongside the emergence of TMEM217 during vertebrate evolution. It is noteworthy that TMEM217 and SLC9C1 are localized in the principal piece of the sperm tail. This colocalization suggests that structural divergence of vertebrate spermatozoa during evolution (41, 42) may have given rise to a mechanism dependent on TMEM217 for proper localization of SLC9C1.

The interaction of TMEM217 with SLC9C1 but not with other NHE family proteins (*SI Appendix,* Table S1) supports the idea that TMEM217 interacts with the VSD, which is unique to SLC9C1 and SLC9C2 (13). In sea urchins, Na^+^/H^+^ exchange mediated by SLC9C1 is regulated by hyperpolarization sensitized via the VSD (17). However, in humans, such hyperpolarization-dependent pH_i_ increase is not observed (43). In mice, pH_i_ increases in response to hyperpolarization have been reported (44, 45), although the specific contribution of the VSD remains unclear. Notably, our study demonstrates that the VSD of SLC9C1 mediates the complex formation with TMEM217, which functions in localizing SLC9C1 to spermatozoa. This finding provides insights into the role of the VSD in protein localization, shedding light on the functional significance of the VSD in SLC9C1.

In addition to SLC9C1, SLC9C2 also contains the VSD among NHE family proteins. SLC9C2 was found to be localized in the sperm heads of humans and rats, but was identified to be a pseudogene in mice (13). We also encountered challenges in analyzing mouse SLC9C2 due to limited annotation in major databases. Further experimental validation and refined annotations would help to clarify the biological roles of mammalian SLC9C2 and its VSD.

Although SLC9C1 is an essential Na^+^/H^+^ exchanger for functional spermatozoa, other family proteins, such as SLC9A1 (NHE1), SLC9A5 (NHE5), SLC9B1 (NHA1), and SLC9B2 (NHA2), are also present in sperm tails (46, 47). A previous study has shown that *Slc9b1* conditional KO mice and *Slc9b2* conditional KO mice are subfertile due to reduced sperm motility, which is accompanied by reduced sAC_fl_ levels and decreased cAMP production in spermatozoa (47). Furthermore, *Slc9b1*/*Slc9b2* double-KO (dKO) male mice were infertile (47). The amino acid sequences of SLC9B1 and SLC9B2 are similar (63.2% similarity, EMBOSS Needle; https://www.ebi.ac.uk/jdispatcher/psa/emboss_needle), suggesting potential compensatory functions between these proteins. Notably, the motility impairment observed in *Slc9b1* KO spermatozoa was rescued by dbcAMP treatment, which is similar to *Slc9c1* KO spermatozoa (47). In our study, SLC9C1 was identified to interact with TMEM217, while neither SLC9B1 nor SLC9B2 was found to interact with TMEM217 (*SI Appendix,* Table S1). SLC9B1 was present in spermatozoa of not only WT mice but also *Tmem217* KO mice (*SI Appendix,* Table S1). On the other hand, SLC9B2 was detected in only one WT mouse, likely due to its low expression levels (*SI Appendix,* Table S1). These results suggest that the absence of SLC9C1 cannot be compensated by SLC9B1, and both SLC9C1 and SLC9B1 may play a role in sAC_fl_-mediated cAMP production independently.

Experiments using *SLC9C1*-overexpressing HEK293T cells indicate that *TMEM217* deletion did not impair SLC9C1 protein levels or its localization to the plasma membrane. These results suggest that SLC9C1 can be localized to the plasma membrane without TMEM217, and SLC9C1 transport to the flagellum may be disrupted in *Tmem217* KO mice. The localization of membrane proteins to cilia involves intraflagellar transport (IFT) (48). We found that proteins involved in intraciliary transport (GO:0042073) were enriched in the TMEM217 interactome, such as IFT122, IFT139 (TTC21B), and IFT121 (WDR35) (Dataset S2)[Bibr r49][Bibr r50]. These proteins are components of the IFT-A complex, which is reported to be essential for the localization of ciliary membrane proteins (48–52). These results suggest that TMEM217 and these IFT proteins may be involved in the flagellar transport and localization of SLC9C1 in male germ cells.

In addition to impaired sperm motility, *Tmem217* KO spermatozoa exhibit flagellar angulation under isotonic conditions (Fig. 3*E*). Defects in ion transport across the sperm plasma membrane due to SLC9C1 depletion may contribute to the flagellar angulation in *Tmem217* KO spermatozoa. However, it has not been reported that *Slc9c1* KO spermatozoa exhibit flagellar angulation (14). Because the osmotic pressure of the medium appears to be critical in flagellar angulation, further analyses with different media may uncover similar flagellar angulation in *Slc9c1* KO spermatozoa. Intriguingly, *Adcy10* KO spermatozoa display flagellar angulation, which is similar to *Tmem217* KO spermatozoa (4). An increase in cAMP regulated by sAC could stimulate ion transporters (53), disruption of which may cause flagellar angulation.

Previous work in sea urchin spermatozoa reported that cAMP production increases upon membrane hyperpolarization, independently of alkalization or Ca^2+^ influx, suggesting that sAC can be activated in a membrane potential-dependent manner (54). In mice, AlphaFold3-based complex models of TMEM217, SLC9C1, and sAC_fl_ predict that the catalytic C1-C2 domains of sAC_fl_ are positioned adjacent to the CNBD within the soluble cytoplasmic domain (CTD) of SLC9C1 (*SI Appendix,* Fig. S9) (55). In sea urchins, hyperpolarization is thought to cause downward movement of the S4 segment in the VSD, leading to large conformational changes of the CTD (15). Although direct structural evidence for voltage-dependent conformational changes in mammals is lacking, such changes could potentially affect the catalytic C1-C2 domains of sAC.

This study underscores the essential role of TMEM217 in regulating SLC9C1 localization in spermatozoa, which is required for sAC/cAMP signaling, sperm motility, and male fertility. Previous MS analyses of human spermatozoa from three different donors detected TMEM217 (43). Furthermore, a *TMEM217* frameshift mutation was found with exome analyses, although its frequency is low in the global population (e.g., 6-37219024-GT-G, 4.96e-6 in total, gnomAD). Further analyses on the TMEM217–SLC9C1–sAC axis may shed light on understanding idiopathic male infertility. In addition, we found that fertilizing ability can be restored in *Tmem217* KO spermatozoa using specialized IVF media including cAMP analogs, which may provide a strategy for overcoming infertility associated with defects in cAMP-dependent pathways.

## Materials and Methods

### Generation of KO Mice.

*Tmem217* KO mice containing a large deletion of the coding region were generated using CRISPR/Cas9-mediated double-strand break. We designed gRNA that recognizes the sequence, (5′-) ATGCTGGGATTGTCACTACG (-3′), upstream to the start codon (gRNA #1), and (5′-) AGCACCACAGTGAATAACGG (-3′), inside the exon 3 (gRNA #2). To generate the gRNA/Cas9 ribonucleoprotein complex, gRNA was mixed with tracrRNA (#TRACRRNA05N-5NMOL, Sigma-Aldrich) and Cas9 (#A36498, Thermo Fisher Scientific). To obtain zygotes, superovulated B6D2F1 females were mated with B6D2F1 males. The gRNA/Cas9 ribonucleoprotein (29.6 ng/µL gRNA, 72.4 ng/µL Cas9) was then introduced into zygotes via electroporation. Electroporated zygotes were cultured in KSOM medium (56) overnight and transferred into the oviducts of the pseudopregnant ICR females. Pups with large deletions at targeted sites were screened by genotyping PCR using primers shown in *SI Appendix,* Table S2 and subsequent Sanger sequencing. The founder mouse was mated with B6D2F1 WT mice to obtain the next generation, and subsequent mating was performed to obtain KO mice.

### Statistical Analysis.

The results were analyzed by GraphPad Prism version 9 (GraphPad software, USA). Data were analyzed using the two-tailed unpaired Student’s *t* test. For a comparison of more than two groups, one-way ANOVA followed by Tukey’s multiple comparisons test was used. Differences were considered significant at *P* < 0.05 (*) or highly significant at *P* < 0.01 (**) and *P* < 0.001 (***). Error bars represent SD.

Extensive experimental procedures are provided in *SI Appendix*, Materials and Methods**.

## Supplementary Material

Appendix 01 (PDF)

Dataset S01 (XLSX)

Dataset S02 (XLSX)

Dataset S03 (XLSX)

Movie S1. Sperm motility of WT mice at 10 min. Sperm motility was videotaped at 50 frames per second 10 min after incubation. The movie is played at 20 frames/second (1/2.5 speed).

Movie S2. Sperm motility of WT mice at 120 min. Sperm motility was videotaped at 50 frames per second 120 min after incubation. The movie is played at 20 frames/second (1/2.5 speed).

Movie S3. Sperm motility of *Tmem217* KO mice at 10 min. Sperm motility was videotaped at 50 frames per second 10 min after incubation. The movie is played at 20 frames/second (1/2.5 speed).

Movie S4. Sperm motility of *Tmem217* KO mice at 120 min. Sperm motility was videotaped at 50 frames per second 120 min after incubation. The movie is played at 20 frames/second (1/2.5 speed).

Movie S5. Sperm motility of *Tmem217* KO mice at 10 min with dbcAMP and IBMX. Sperm motility was videotaped at 50 frames per second 10 min after incubation with dbcAMP and IBMX. The movie is played at 20 frames/second (1/2.5 speed).

Movie S6. Sperm motility of *Tmem217* KO mice at 120 min with dbcAMP and IBMX. Sperm motility was videotaped at 50 frames per second 120 min after incubation with dbcAMP and IBMX. The movie is played at 20 frames/second (1/2.5 speed).

## Data Availability

All study data are included in the article and/or supporting information.
