# Supplementary material for: Formation of a complex between TMEM217 and the sodium-proton exchanger SLC9C1 is crucial for mouse sperm motility and male fertility
Source: Proc Natl Acad Sci U S A. 2025 Oct 15;122(42):e2513924122. doi: 10.1073/pnas.2513924122 (PMC12557800; doi:10.1073/pnas.2513924122)
Supplement: Supplementary file 1 — Appendix 01 (PDF) [file pnas.2513924122.sapp.pdf]

PNAS

Supporting Information for
Formation of a complex between TMEM217 and the sodium-proton exchanger SLC9C1 is crucial for mouse sperm motility and male fertility

Rie Iida-Norita[a,b], Haruhiko Miyata[a*], Akinori Ninomiya[a], Chihiro Emori[a], Maki Kamoshita[a], Chen Pan[a,c], Haoting Wang[a,c], and Masahito Ikawa[a,b,c,d,e,f*]

[a]Department of Experimental Genome Research, Research Institute for Microbial Diseases (RIMD), The University of Osaka, 3-1 Yamadaoka, Suita, Osaka 565-0871, Japan

[b]Animal Resource Center for Infectious Diseases, Immunology Frontier Research Center (IFReC), The University of Osaka, 3-1 Yamadaoka, Suita, Osaka, 565-0871, Japan

[c]Laboratory of Experimental Genome Research, Graduate School of Pharmaceutical Sciences, The University of Osaka, 1-6 Yamadaoka, Suita, Osaka 565-0871, Japan

[d]Division of Microbiology and Immunology, Center for Infectious Disease Education and Research (CiDER), The University of Osaka, 1-10 Yamadaoka, Suita, Osaka, 565-0871, Japan

[e]Team of Vaccine Evaluation, Center for Advanced Modalities and DDS, The University of Osaka, 2-8 Yamadaoka, Suita, Osaka 565-0871, Japan

[f]Laboratory of Reproductive Systems Biology, The Institute of Medical Science, The University of Tokyo, 4-6-1 Shirokanedai, Minato-ku, Tokyo 108-8639, Japan

[*]Corresponding authors: Haruhiko Miyata and Masahito Ikawa

Email: hmiya003@biken.osaka-u.ac.jp (H.M.); ikawa@biken.osaka-u.ac.jp (M.I.)

This PDF file includes:

SI Materials and Methods
Figures S1 to S9
Tables S1, S2
Legends for Movies S1 to S6
Legends for Datasets S1 to S3
SI References

Other supporting materials for this manuscript include the following:

Movies S1 to S6
Datasets S1 to S3

SI Materials and Methods

Animals and ethics

Mice were purchased from Japan SLC or CLEA Japan, kept in an environment free of specific pathogens, followed a 12-hour light/12-hour dark cycle, and given free access to water and food. All animal experiments were approved by the Animal Care and Use Committee of the Research Institute for Microbial Diseases, Osaka University (#Biken-AP-R03-01; approval date: May 19, 2021, in Osaka University). This study was performed in accordance with ARRIVE guidelines 2.0 (https://arriveguidelines.org/arrive-guidelines). Frozen spermatozoa of KO and Tg mice generated in this study will be made available at the RIKEN BioResource Research Center and the Center for Animal Resources and Development, Kumamoto University.

Generation of Tg mice

The p*Clgn*-mouse *Tmem217-3xFLAG* construct was generated as described below. Linearized DNA with SacI and PacI was injected into two pronuclei (2PN) zygotes obtained by *in vitro* fertilization using *Tmem217* heterozygous mice. As with the generation of KO mice, the injected zygotes were cultured and implanted into pseudopregnant female mice.

Isolation of testicular germ cells (TGC)

Isolation of TGC was performed according to a previous study with some modifications (1). Briefly, the seminiferous tubules were minced with a razor blade to release germ cells. The cell suspension in PBS with 1 mM EDTA was filtered through a 70 µm nylon mesh, collected by centrifugation at 600 × *g* for 5 min, and subjected to immunoblot analysis either directly or after the isolation of testicular spermatozoa.

Phase separation of Triton X-114 extracts of TGC

Phase separation of Triton X-114 extracts of TGC was performed according to a previous study (2).

Immunostaining of spermatozoa

Spermatozoa were collected from the cauda epididymis and suspended in ice-cold PBS. Sperm suspension was pipetted onto MAS coat slides (#TF1006M, MATSUNAMI), left at 4°C for 30 min for the attachment of the spermatozoa to the slide, fixed with 4% paraformaldehyde in PBS for 10 min, and permeabilized with 0.1% Triton X-100 in PBS for 10 min. After overnight incubation with anti-FLAG antibody (#M185-3L, dilution 1:100, MBL) at 4 °C, spermatozoa were incubated with Alexa Fluor 546 anti-mouse IgG for 1 h followed by Hoechst 33342 (#62249, dilution 1:5000, Invitrogen) for 10 min, and mounted with Immu-Mount (#9990402, Thermo Fisher Scientific). The slides were observed using a BX53 microscope (Olympus).

Immunoprecipitation

Testes were lysed with 1% Triton X-100 in TBS and centrifuged at 20,000 × *g* for 10 min. The lysates were incubated for 2 h at 4 °C with anti-FLAG antibody (#F1804, Sigma-Aldrich)-conjugated Dynabeads (#10009D, Thermo Fisher Scientific). The immune complexes were washed three times with an IP buffer containing 40 mM Tris-HCl, 150 mM NaCl, 0.1% Triton X-100, and 10% glycerol and eluted by incubation with SDS sample buffer for 10 min at 75 °C. Eluates were then used for immunoblot or MS analysis.

For cultured cells, cells were lysed with Pierce IP lysis buffer (#87787, Thermo Fisher Scientific) and centrifuged at 13,000 × *g* for 10 min. At least 100 µg of lysates was incubated for 1 h

at 4 °C with anti-FLAG antibody (#F1804, Sigma-Aldrich) or anti-PA antibody (#012-25841, Fujifilm WAKO)-conjugated Dynabeads. The immune complexes were washed three times with IP buffer and eluted by incubation with 3xFLAG peptide (#F3290, Sigma Aldrich) or PA peptide (#161-28681, Fujifilm Wako) diluted with Pierce IP lysis buffer.

Analysis of sperm motility

Sperm motility was analyzed as described previously (2). Spermatozoa collected from the cauda epididymis were incubated in a drop of TYH medium (3) with or without 5 mM dbcAMP (#S7858, Selleck) and 100 µM IBMX (#S5836, Selleck) at 37 °C under 5% CO_2 in air. For examination of sperm motility, sperm suspension taken from the top of the TYH drop was analyzed with the CEROS II sperm analysis system (software version 1.5; Hamilton Thorne Biosciences). For capturing movies, spermatozoa were observed with an Olympus BX-53 microscope equipped with a high-speed camera (HAS-L1, Ditect). The motility was videotaped at 50 frames per second.

Detection of phospho-tyrosine and phospho-PKA-substrates in spermatozoa

Spermatozoa were incubated in the same conditions used for the motility analysis. Samples were prepared according to a previous report (4) with some modifications. TYH drops containing spermatozoa were mixed with 1 ml PBS containing phosphatase inhibitor (#07574-61, Nacalai Tesque) on ice, and centrifuged at 2,000 × *g* for 5 min at 4 °C to collect spermatozoa. The obtained spermatozoa were resuspended in sample buffer without β-mercaptoethanol and boiled for 5 min. After centrifugation, 5% β-mercaptoethanol was added to the supernatants, and the mixture was boiled again for 1 min. Denatured samples were subjected to immunoblotting.

***In vivo* fertility**

Sexually mature WT or *Tmem217* KO males were housed individually with three 8-week-old B6D2F1 female mice for at least 8 weeks. Male mice were removed after the mating period, and females were kept for another 3 weeks to count the final litters. The number of pups and copulation plugs was counted every weekday morning. For analyzing the fertility of *Tmem217* KO; Tg mice, sexually mature male mice were housed individually with three 8-week-old B6D2F1 female mice for 8 weeks, and the litter size was recorded during this period.

***In vitro* fertilization**

In vitro fertilization (IVF) was performed as previously described (5). Oocytes were collected from the ampulla of superovulated B6D2F1 female mice and cultured in a drop of 100 µL TYH medium. Spermatozoa obtained from WT or *Tmem217* KO mice were incubated in a drop of 100 µL TYH medium for 120 min at 37 °C under 5% CO_2 in air to induce capacitation (preincubation). Spermatozoa were then obtained from the top of the drop and were incubated with oocytes at a final density of 2×10^5 spermatozoa/mL (insemination). The formation of PN was observed 6 h after insemination.

To rescue *Tmem217* KO fertility by dbcAMP and IBMX, oocytes from WT female mice were cultured in a drop of 100 µL TYH or CARD MEDIUM (Kyudo Co., Ltd). Spermatozoa obtained from WT or *Tmem217* KO mice were incubated in a drop of TYH or CARD FERTIUP Preincubation Medium (Kyudo Co., Ltd) with or without 5 mM dbcAMP (Selleck) and 100 µM IBMX (Selleck) at 37°C under 5% CO_2 in air. Insemination was performed as described above. The oocytes were collected 3 h after insemination and cultured in KSOM. The formation of PN was observed 6 h after insemination. The next day, 2-cell embryos were transplanted into the oviduct ampulla of pseudopregnant ICR females. Pups were counted and genotyped one week after birth.

Isolation of mouse spermatocytes and round spermatids

Spermatocytes were isolated by fluorescence-activated cell sorting as previously described with minor modifications (6). Briefly, the testes of an individual adult mouse were collected in Gey's Balanced Salt Solution (GBSS) (#G9779, Sigma Aldrich). The tunica albuginea was removed, and the seminiferous tubules were digested with collagenase (#C5138, 50 µg/ml, Sigma Aldrich) and DNase I (#DN25, 2.5 µg/ml, Sigma Aldrich) at 36 °C with gentle agitation for 20 min and digested with 0.25% trypsin and DNase I (2.5 µg/ml) at room temperature for 20 min. The digestion was terminated with 5% fetal bovine serum. The suspension was then filtered through a 40 µm cellular filter, and the flow-through was centrifuged at 190 × *g* for 5 min to collect the cell pellet. The cells were resuspended in GBSS containing 10% FBS and stained with Hoechst 33342 (10 µg/ml, Life Technologies) under shaking at 32 °C for 20 min, and spermatocytes and round spermatids were sorted by a BD FACSymphony S6 (BD Biosciences) coupled with an ultraviolet laser (355 nm), using a 100-micron nozzle. Cells were gated according to their scatter (FSC versus SSC) parameters; fluorescence of Hoechst 33342 was measured on non-aggregated cells. Hoechst red (740/35 bandpass filter) versus Hoechst blue (450/50 bandpass filter) from the UV laser were used on a dot plot in order to discriminate populations.

Preparation of testicular and epididymal spermatozoa

Following previous reports, TGCs were suspended in 52% isotonic Percoll (#17089102, GE Healthcare) and centrifuged for 30 min (38,000 × *g*, 4 °C) using Optima XPN (Beckman), and testicular spermatozoa were isolated (7, 8). Caput and cauda epididymis were minced and filtered through a 70 µm nylon mesh. Cells were then collected by centrifugation at 600 x *g* for 5 min.

Quantification of intracellular cAMP

Spermatozoa collected from the cauda epididymis were incubated in a drop of TYH medium at 37 °C under 5% CO_2 in air. Sperm number in the drop was counted and the suspension was centrifuged at 2,000 × *g* for 2 min. Collected spermatozoa were immediately stored at -80 °C. Then, samples were incubated in 0.1 M HCl for 30 min, mixed with a pipette, and centrifuged at 1,000 g for 10 min. Supernatants were collected and subjected to quantification analysis of cAMP with Cyclic AMP ELISA Kit (#581001, CAYMAN) using the acetylation method according to the manufacturer's instructions.

Cell culture and transfection

HEK293T cells (9) were maintained in DMEM (#11965092, Gibco) with 10% FBS (#F7524, Sigma-Aldrich) and penicillin-streptomycin (#10378-016, Gibco). Cells were maintained at 37°C in a 5% CO_2 humidified air atmosphere. HEK293T cells were seeded 1 x 10^6 per well in a 6-well plate, and transfected by the calcium phosphate–DNA co-precipitation method. After 24 h or 48 h incubation with reagents, cells were subjected to subsequent assays.

Plasmids

Coding sequences of mouse *Tmem217* (NM_001162901), mouse *Slc9c1*(NM_198106), and human *TMEM217* (NM_001286401) were cloned and amplified by PCR using cDNA obtained from adult C57BL/6 mouse testis or human testis (#637209, Clontech). PCR amplicon was inserted into the cloning site of pCAG1.1 vector (Addgene; Plasmid #173685). Mouse *Tmem217* was also inserted into the cloning site of *pClgn* vector (Addgene; Plasmid #173686), which contained a Calmegin promoter followed by a C-terminal 3xFLAG-tag with poly(A) signal. Human *SLC9C1* vector was purchased from Addgene (pDONR221_SLC9C1, Addgene; #132247) and subcloned into the pCAG1.1 vector. Primers used for PCR are listed in Table S2.

Establishment of *TMEM217* KO cell lines

The gRNA sequences targeting human *TMEM217* 5′-GAACAGAAGCACCTAGGGAA -3′ (gRNA #1) and 5′-CAAGAGACGAATTTCTACAG -3′ (gRNA #2) were designed inside the coding region as shown in Fig.S7. These sequences were separately inserted into pSpCas9(BB)-2A-Puro (PX459; Addgene plasmid #48139) and co-transfected into HEK293T cells following the protocol described above. After 48 h of transfection, transfected cells were selected in medium containing 1.4 μg/ml puromycin, and the medium was changed every 2 or 3 days for selection of positive clones. Two weeks post-transfection, the positive clones were subjected to limiting dilutions in 96-well plates for selection of monoclonal cells. *TMEM217* KO cell lines were obtained and confirmed by RT-PCR using primers listed in Table S2.

Immunostaining of HEK293T cells

HEK293T cells were seeded on coverslips in a 6-well plate at a density of 2 × 10^5 cells and cultured for 6–16 h at 37 °C in DMEM with 10% FBS. Human *SLC9C1-3xFLAG* and *TMEM217-PA* expression vectors were transfected as described above. After 24 h, the medium was changed to serum-free medium and further incubated for 48 h. Then, cells were fixed with 4% paraformaldehyde in PBS for 15 min, permeabilized with 0.1% Triton X-100 for 10 min, blocked with 10% FBS for 1 h, and incubated with anti-FLAG antibody (#M185-3L, dilution 1:1000, MBL) overnight at 4 °C. Cells were then incubated with Alexa Fluor 488 anti-mouse IgG antibody (#A32723, dilution 1:1000, Invitrogen) followed by incubation with Hoechst 33342 and mounted as described above. The slides were observed using a Nikon Eclipse Ti microscope connected to a Nikon C2 confocal module.

RT-PCR

Reverse transcription of multiple tissue samples from C57BL/6N mice was performed according to a previous report (2). PCR was then performed using 10 ng cDNA. For HEK293T cells, Buffer RLT of the RNeasy mini kit (#74104, Qiagen) was used for harvesting cells, and RNA was extracted according to the manufacturer's protocol of the kit. PCR was then performed using 250 ng cDNA. Primers used for PCR are listed in Table S2.

Immunoblot analysis

Testis, spermatozoa, and cultured cells were lysed with Pierce IP lysis buffer (#87787, Thermo Fisher Scientific) containing 0.1% sodium dodecylsulfate (SDS) and protease inhibitor (Nacalai Tesque). After homogenization, the lysates were centrifuged at 13,000 × *g* for 10 min to collect supernatants. The supernatant was mixed with SDS sample buffer and incubated at 95 °C for 5 min; for membrane proteins that tended to aggregate at high temperatures, samples were incubated in SDS sample buffer at 4 °C for 1 h or overnight instead. Blots were blocked with 10% skim milk in Tris-buffered saline containing 0.1% Tween 20 (TBST) and incubated with rabbit anti-SLC9C1 antibody (0.6 μg/ml, generated against amino acid residues 1158-1175 with modification of cysteine at N-terminus of the peptide [CGKKENQENEELIEENINI]), mouse sAC antibody (#MABS2257, clone R21, dilution 1:100, Sigma Aldrich), rat anti-PA antibody (#012-25863, Fujifilm WAKO), rabbit anti-FLAG antibody (#PM020, dilution 1:1000, MBL), mouse anti-α-tubulin antibody (#T6074, dilution 1:5000, Sigma Aldrich), mouse anti-phospho-tyrosine antibody (#05-1050, dilution 1:1000, 4G10, Sigma-Aldrich), rabbit anti-phospho-PKA Substrate (RRXS*/T*) antibody (#9624, dilution 1:1000, 100G7E, Cell Signaling Technology), overnight at 4 °C. After three washes in TBST, the blots were incubated with appropriate secondary antibodies conjugated to horseradish peroxidase (Jackson ImmunoResearch) for 2 h at room temperature. After additional three washes in TBST, the bands were detected with Amersham ImageQuant 800

(Cytiva, Osaka, Japan) using Chemi-Lumi One Super or Chemi-Lumi One Ultra (Nacalai Tesque).

HePAS staining

Testes were fixed with Bouin's fixative, dehydrated in ethanol, embedded in paraffin, and cut with a microtome. The sections were stained with hematoxylin and periodic acid Schiff (HePAS) as described previously (10).

Measurement of the number of bent spermatozoa

For analysis of sperm morphology at different osmolality conditions, TYH medium was prepared as 400, 314, and 150 mOsm solutions by adding the appropriate NaCl concentration. Spermatozoa from the cauda epididymis were incubated in TYH for 30 min at 37 °C under 5% CO_2 in air, followed by incubation with 10 μg/ml propidium iodide (PI) for 5 min. Spermatozoa were observed and counted using an Olympus BX-53 microscope (Olympus). The shapes of their tails were classified as normal (straight) or bent (hairpin-like). At least 100 spermatozoa were counted for each trial.

NanoLC-MS/MS analysis of testis

Immunoprecipitates from testis lysates were subjected to nanoLC-MS/MS analysis as described previously (11). The tryptic peptides were separated using a nanoElute (Bruker Daltonics) with a PepSep C18 column (10 cm × 75 μm, 1.9 μm, Bruker Daltonics), and the eluted peptides were analyzed using timsTOF Pro (Bruker Daltonics). The resulting data were processed using DataAnalysis (Bruker Daltonics), and proteins were identified using MASCOT Server 2.7.0 (Matrix Science) and the UniProt mouse database. Quantitative values were calculated by Scaffold 5 software (Proteome Software).

Gene Ontology (GO) enrichment analysis of TMEM217 interacting proteins was performed with clusterProfiler (12). *P* values were adjusted by the Benjamini-Hochberg method. The top 15 terms by adjusted *P* values were visualized with bar plots.

NanoLC-MS/MS analysis of mature spermatozoa lysate

Mature spermatozoa were lysed with 1% Triton X-100 in TBS, rotated for 2 h at 4 °C, and centrifuged at 20,000 × *g* for 10 min. The lysates were purified by methanol and chloroform, dissolved in 0.1% RapiGest (Waters), reduced with 10 mM DTT, alkylated with 55 mM iodoacetamide, digested with trypsin (Promega), and purified with a C18 tip (AMR). The tryptic peptides were fractionated into eight fractions using a SCX stage tip (#7510-11203, GL Science). Eight samples from the first fraction were purified with the C18 tip (C-tip, AMR), dried by centrifuge concentrator, and dissolved in a 0.1% aqueous solution of formic acid (FA). The fractionated samples were analyzed by a nanoLC-MS/MS system as described above, and a C18 capillary column (75 μm x 125 mm, 1.9 μm, Nikkyo Technos) was employed for peptide separation. The peptides were eluted using solvent A (0.1% FA in water) and solvent B (0.1% FA in acetonitrile) by linear gradient of 2-35% B for 20 min at 250 nL/min. The eluted peptides were analyzed on a timsTOF Pro and measured in diaPASEF mode. The instrument parameters were the default setting of dia PASEF short gradient mode. Briefly, the source parameters were as follows: capillary voltage, 1,600 V; dry gas, 3.0 L/min; and dry temperature, 180 °C. The MS1 and MS2 spectra were collected in the *m/z* range of 100–1,700. The accumulation and ramp times were set to 100 ms. The dia PASEF window ranged in dimensions from *m/z* 475 to 1,000 and in dimension 1/K0 from 0.85–1.27. DIA-MS files were searched against an *in silico* mouse spectral library using DIA-NN (version 1.8.1)(13). First, a spectral library was generated from the UniProt mouse and human protein sequence database using DIA-NN. The parameters for

generating the spectral library were as follows: digestion enzyme, trypsin; missed cleavage, 1; peptide length range, 7–30; precursor charge range, 1–4; precursor *m/z* range, 300–1,800; and fragment ion *m/z* range, 200–1,800. “FASTA digest for library-free search/library generation;” “deep learning-based spectra, RTs, and IMs prediction;” “n-term M excision;” and “C carbamidomethylation” were enabled. DIA-NN search parameters were as follows: mass and MS1 accuracy, optimized the mass accuracy automatically using the first run in the experiment; and protein inference, genes, off. The MBR was turned on for quantitative analyses of the proteins and precursors. The protein identification threshold was set at 1% or less for both precursor and protein FDRs. When reporting protein numbers and quantities, the ‘Protein.Group’ column in DIA-NN’s report was used to identify the protein group. The MaxLFQ column (calculated using the MaxLFQ algorithm) was used to obtain the normalized quantity (14). The normalized quantity was converted to log2 and statistically analyzed using R software. The data was visualized using the pheatmap (15)(https://github.com/raivokolde/pheatmap) and EnhancedVolcano R package (https://github.com/kevinblighe/EnhancedVolcano) to illustrate differentially expressed proteins.

***In silico* data analyses**
The degree of co-evolution was evaluated using the CladeOScope (https://tabachlab.shinyapps.io/CladeOScope/) (16). Amino acid sequences of TMEM217 were aligned using Clustal Omega (https://www.ebi.ac.uk/Tools/msa/clustalo/). The aligned sequences were manually curated and visualized using Jalview (version 2.11.2.0, https://www.jalview.org/). TMEM217 amino acid sequences were obtained from UniProt for human (Q8N7C4), cynomolgus monkey (A0A2K5V5G5), bovine (Q32LD5), pig (F1RVS4), mouse (Q14AF1), rat (M0RD60), snapping turtle (A0A8T1T3E9), corn snake (A0A6P9BR81), Atlantic salmon (A0A1S3MBT6), Tasmanian devil (A0A7N4P423), and Chinese softshell turtle (K7EYW7). The sequence of West African lungfish (XP_043938011.1) was retrieved from NCBI. SLC9C1 sequences were obtained from UniProt for mouse (Q6UJY2), human (Q4G0N8), bovine (F1MQF9), Tasmanian devil (G3VD84), and purple sea urchin (A3RL54). The sequence of the Chinese softshell turtle (XP_025044710.1) and West African lungfish (XP_043921995.1) were retrieved from NCBI. TMEM217 domains within the sequences were identified using the SMART tool (http://smart.embl-heidelberg.de/). SLC9C1 domains within the sequence were based on the previous report (17). Single-cell transcriptomic data from mouse and human testes were obtained from a previously published study (18). Gene expression profiles across various cell populations were analyzed using the Loupe Cell Browser (version 3.3.1, 10x Genomics). 3D structure of TMEM217-SLC9C1 complex was predicted by AlphaFold3 (https://alphafoldserver.com/) (19) and visualized with the UCSF ChimeraX (https://www.rbvi.ucsf.edu/chimerax) (20).

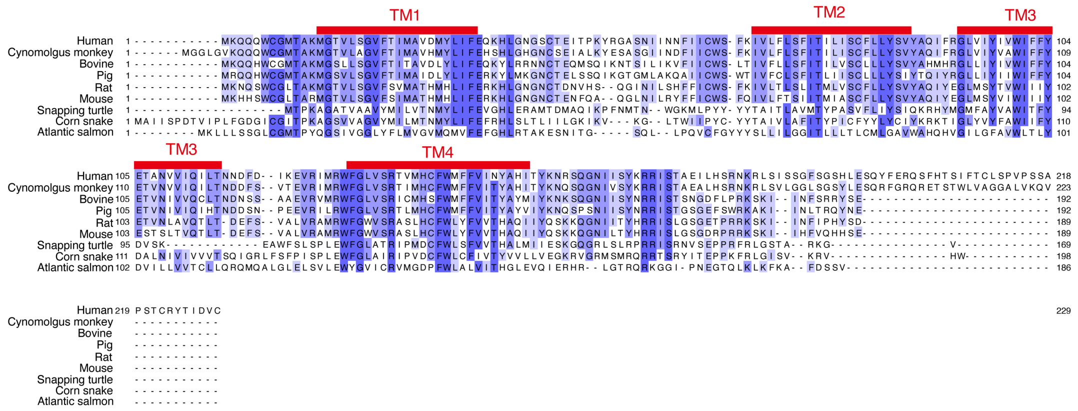

Fig. S1. Amino acid sequence alignment of vertebrate TMEM217.
Amino acid sequences of TMEM217 from each of the indicated species are shown in the alignment. TMs indicate transmembrane domains. Conserved amino acids are colored with the gradation following the degree of conservation among indicated species.

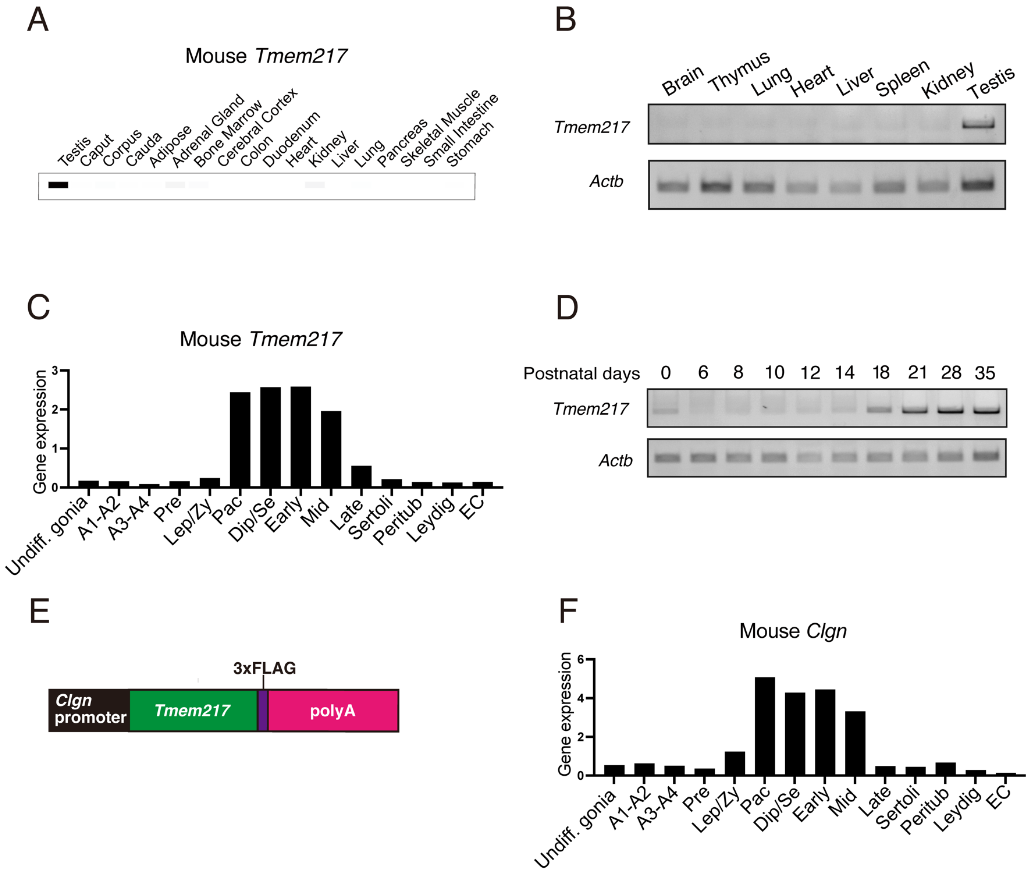

Fig. S2. Expression patterns of *Tmem217*.
(A) Expression patterns of *Tmem217* in mouse tissues. Band intensities are based on transcripts per million (TPM). White = 0 TPM, Black ≥ 30 TPM. (B) RT-PCR analysis of *Tmem217* expression in various mouse tissues. *Actb* was used as a reference gene. (C) Expression profile of mouse *Tmem217* in testicular cells obtained from previously published datasets. Undiff. gonia: Undifferentiated spermatogonia, A1-A2: A1 and A2 differentiating spermatogonia, A3-A4: A3 and A4 differentiating spermatogonia, Pre: preleptotene spermatocytes, Lep/Zy: leptotene and zygotene spermatocytes, Pac: pachytene spermatocytes, Dip/Se: diplotene and secondary spermatocytes, Early: early round spermatids, Mid: mid round spermatids, Late: late round spermatids, Peritub: peritubular myoid cells, EC: Endothelial cells. (D) RT-PCR analysis of *Tmem217* expression in mouse postnatal testes. *Actb* was used as a reference gene. (E) Schematic illustration of the transgene used to generate *Tmem217* Tg mice. (F) Expression profile of mouse *Calmegin* (*Clgn*) in testicular cells obtained from previously published datasets.

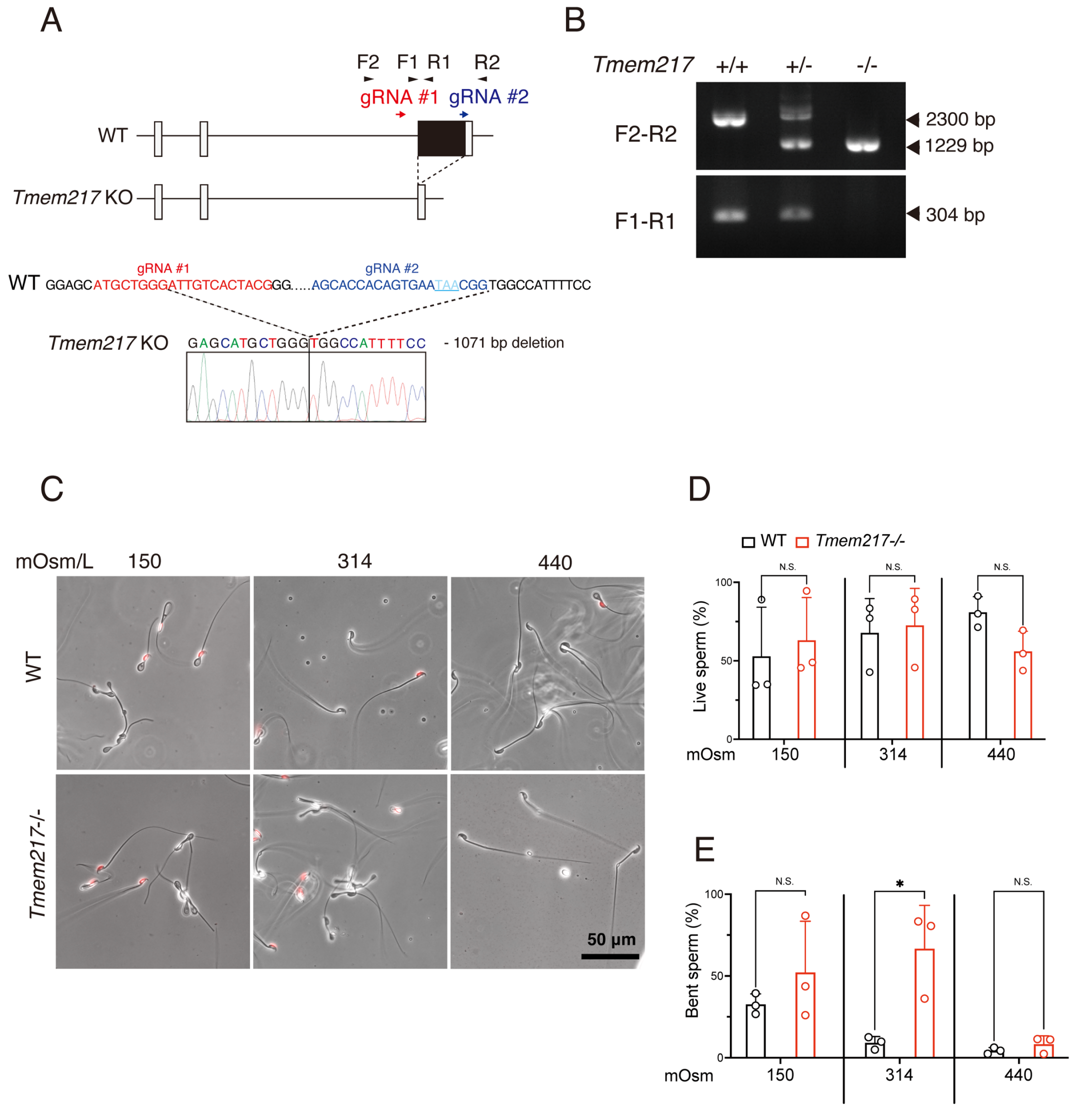

Fig. S3. Generation of *Tmem217* KO mice and analyses of sperm morphology.
(A) Generation of *Tmem217* KO mice using the CRISPR/Cas9 system. We designed gRNA#1 at introns upstream of the coding exon and gRNA#2 at the end of the coding exon including the stop codon (TAA). Forward (F) and reverse (R) primers were used for genotyping. (B) Genotyping PCR for detecting WT and *Tmem217* KO alleles using primers shown in (A) and Table S2. (C)-(E) The hypoosmotic tolerance of sperm tails in WT and *Tmem217* KO mice. Cauda epididymal spermatozoa were released into different osmotic environments (mOsm/L). Microscopic observation of bending sperm tails (C). Dead spermatozoa were detected with propidium iodide (PI). Percentages of live spermatozoa (PI negative) in total Hoechst 33342-positive spermatozoa under different osmotic environments (D). Percentages of tail-bending spermatozoa (E). n = 3 males for each data. Error bars represent SD. *P* value was determined by two-tailed unpaired Student's t-test. $P < 0.05$ (*).

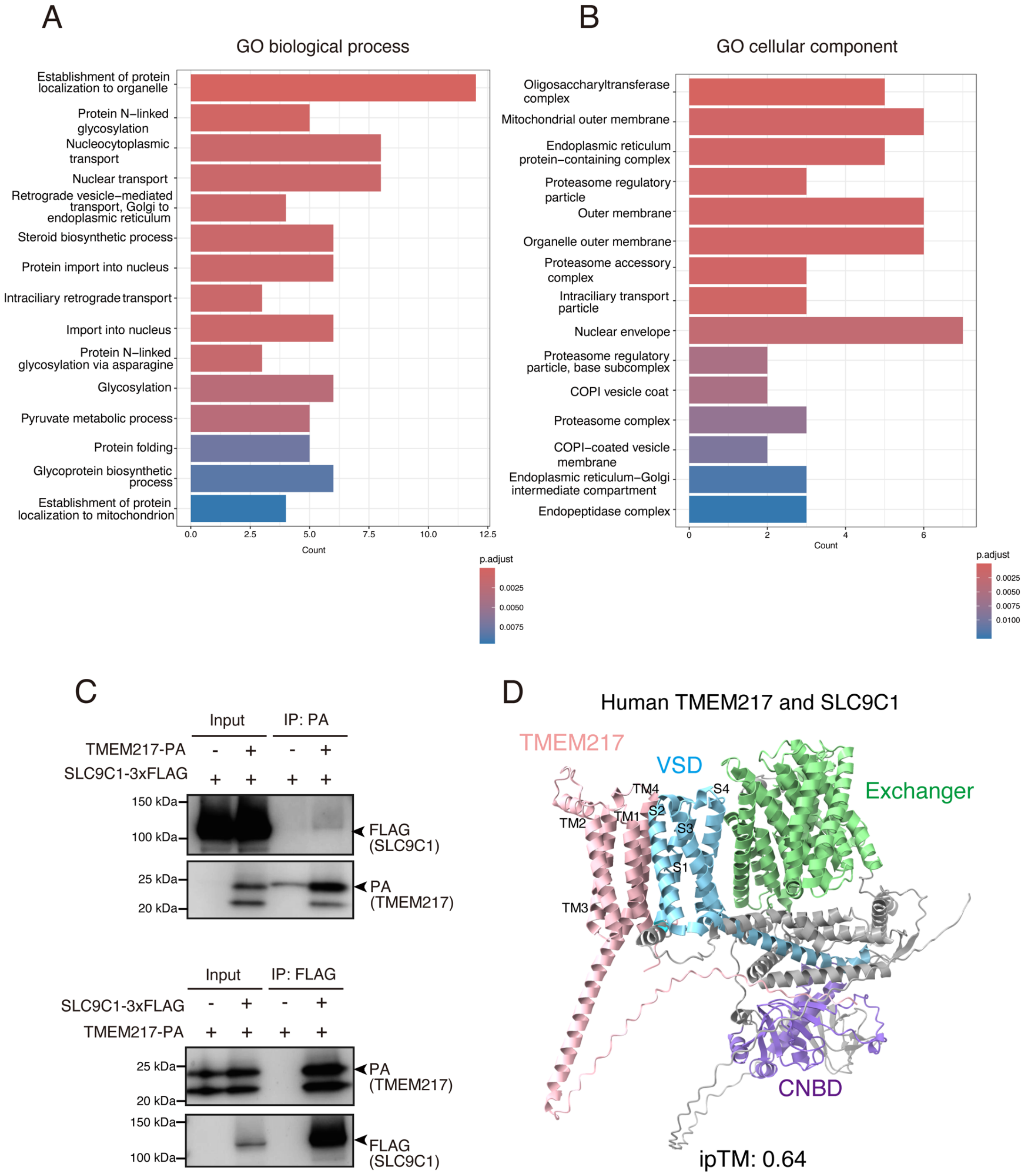

Fig. S4. Co-immunoprecipitation and mass spectrometry analyses of TMEM217.
(A), (B) Top 15 enriched Gene Ontology (GO) terms of TMEM217 interactors in testes. Proteins identified in three Tg lines but not in WT were considered to be TMEM217 interactors. GO terms for biological process (A) and for cellular component (B) are shown. Bar length indicates the number of interactors. Color denotes the adjusted *P* value (p.adjust). (C) Co-immunoprecipitation (IP) analysis using anti-PA (upper panel) and anti-FLAG (lower panel) antibodies on HEK293T cell lysates expressing human TMEM217-PA and SLC9C1-3xFLAG. (D) AlphaFold3 3D structural prediction of the human TMEM217-SLC9C1 complex. The ipTM score of the complex is shown.

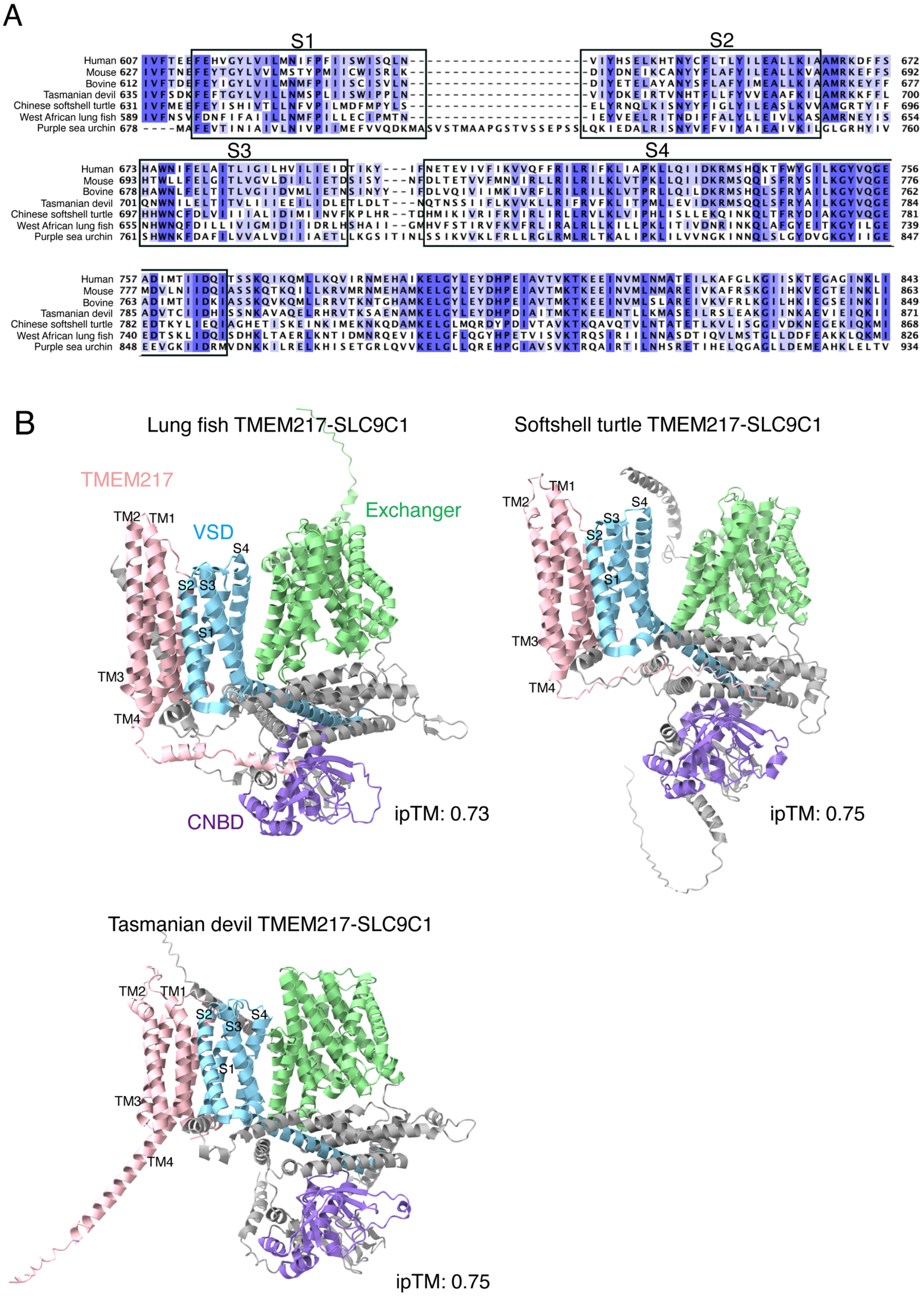

Fig. S5. Analysis of VSD-mediated SLC9C1–TMEM217 interaction in vertebrates.
(A) Amino acid sequence alignment of the VSD region of SLC9C1 from the indicated vertebrate species in (B) and sea urchin. The S1–S4 of the VSD are outlined. Conserved amino acids are colored with the gradation following the degree of conservation among indicated species.

(B) AlphaFold3-predicted 3D structural models of the TMEM217–SLC9C1 complex in lungfish, softshell turtle, and Tasmanian devil. Transmembrane helices TM1-TM4 of TMEM217 and S1–S4 of the VSD are labeled. The ipTM scores of each complex are shown.

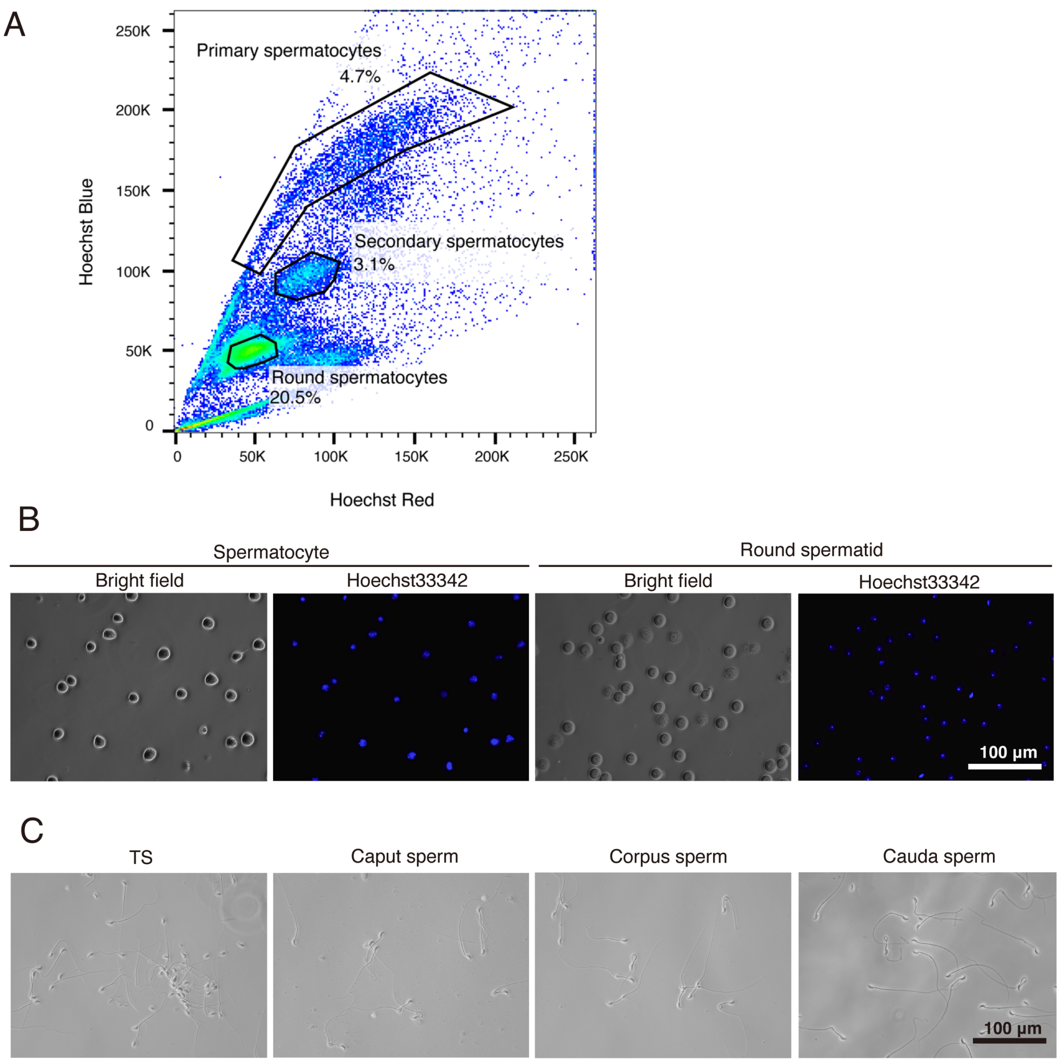

Fig. S6. Separation of germ cells.
(A) Representative FACS 2D plot used for the isolation of spermatocytes and round spermatids. Testicular cells were stained with Hoechst 33342 and analyzed for Hoechst Blue and Hoechst Red fluorescence. Gates were defined to identify primary spermatocytes, secondary spermatocytes, and round spermatids, based on Hoechst fluorescence profiles. Primary and secondary spermatocytes were collected together as "spermatocytes". (B) Representative images of each fraction (spermatocytes, round spermatids) to confirm the purity of the samples used in Fig. 5E. Spermatocytes were characterized by a moderate and distributed Hoechst signal, without distinct chromocenter. Round spermatids were characterized by a distinct Hoechst-bright chromocenter surrounded by euchromatin. (C) Representative images of each fraction [testicular spermatozoa (TS), and spermatozoa from caput, corpus, and cauda epididymis] to confirm the purity of the samples used in Fig. 5F.

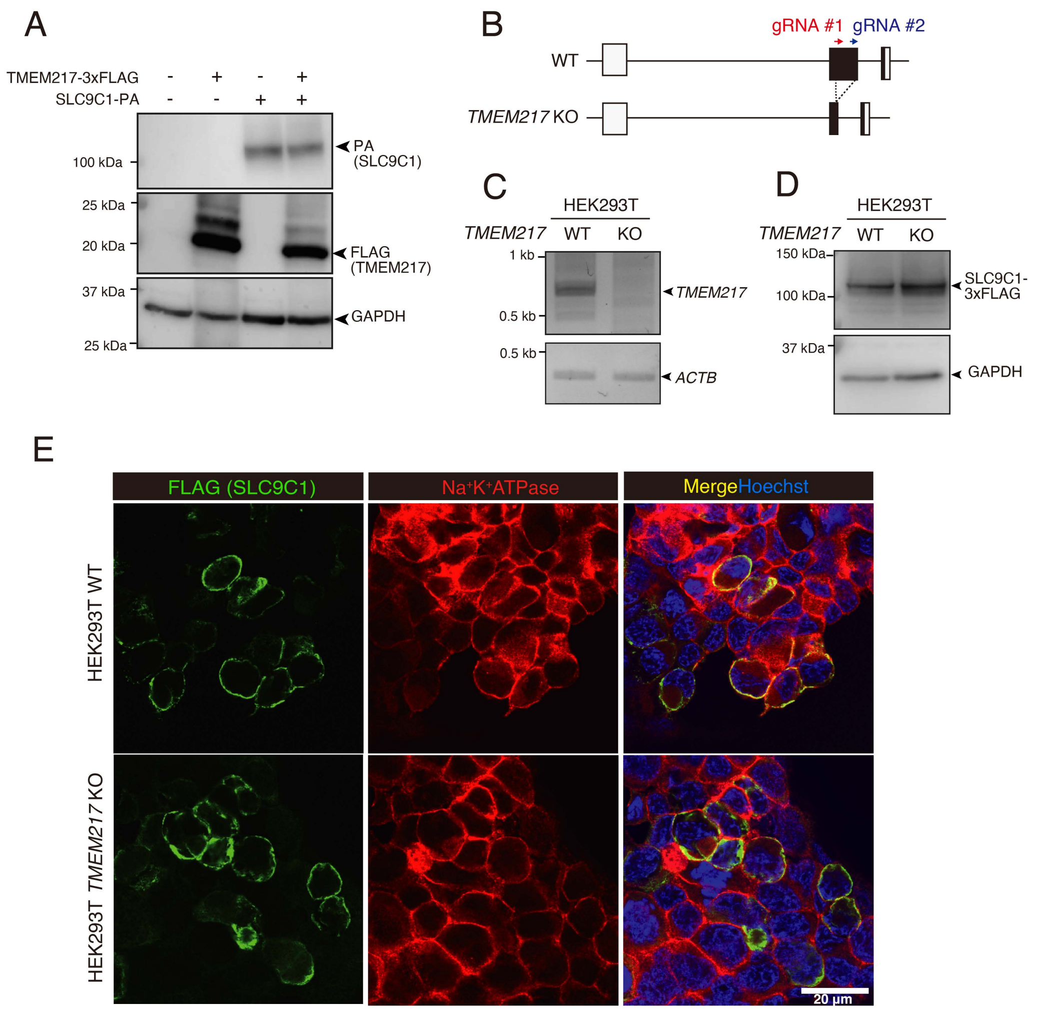

Fig. S7. Analyses of TMEM217 using HEK293T cells.
(A) Immunoblot analysis of HEK293T cells expressing mouse SLC9C1-PA and/or TMEM217-3xFLAG. GAPDH was used as a loading control. (B) Generation of *TMEM217* KO HEK293T cells using the CRISPR/Cas9 system. We designed gRNA#1 at the beginning of the coding exon and gRNA#2 at the end of the coding exon. (C) RT-PCR analysis using primers designed to amplify the full-length TMEM217-coding sequence to evaluate *TMEM217* expression in WT and *TMEM217* KO HEK293T cells. *ACTB* was used as a reference gene. (D) Immunoblot analysis using an anti-FLAG antibody to detect human SLC9C1-3xFLAG in lysates obtained from WT and *TMEM217* KO HEK293T cells. GAPDH was used as a loading control. (E) Immunofluorescence analysis using an anti-FLAG antibody to localize SLC9C1-3xFLAG (green) in WT and *TMEM217* KO HEK293T cells. Na^+/K^+-ATPase was used as a plasma membrane marker (red). Nuclei were stained with Hoechst 33342 (blue).

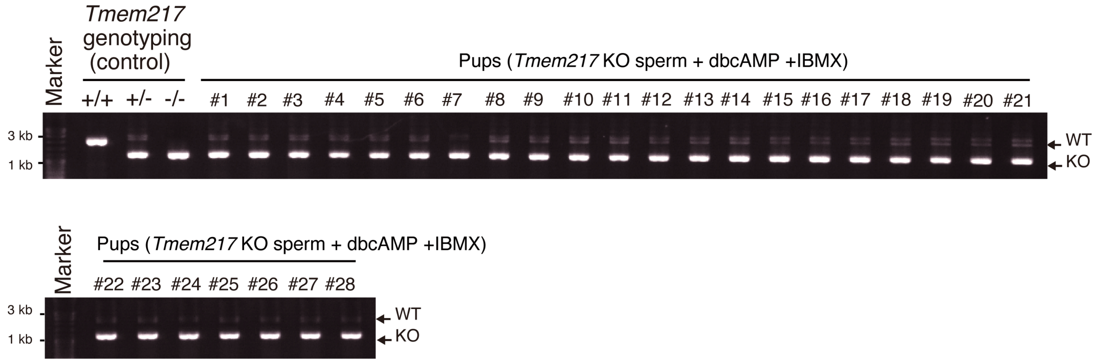

Fig. S8. Genotypes of pups obtained via IVF and embryo transfer.
PCR analysis to detect *Tmem217* KO allele in all pups (#1–#28) obtained via IVF and embryo transfer in Fig. 6H. All pups were confirmed to be heterozygous.

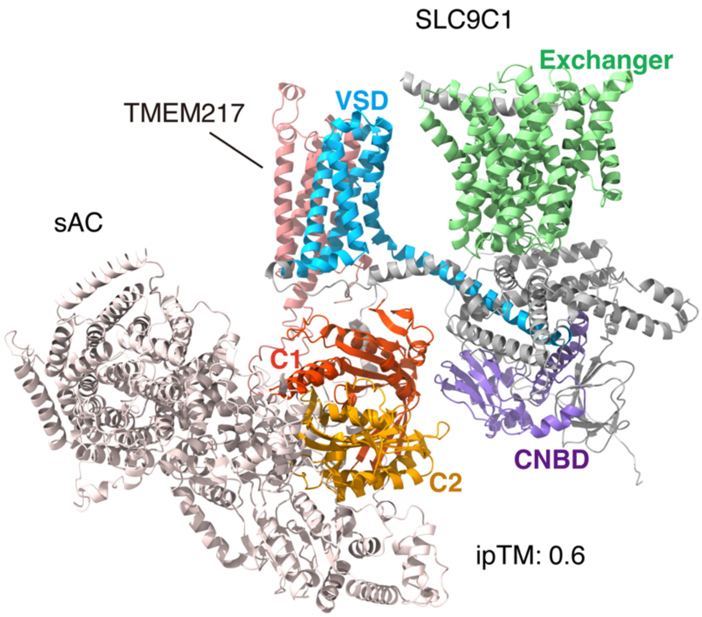

Fig. S9. Structural analysis of the TMEM217–SLC9C1–sAC complex.
AlphaFold3 complex prediction reveals the spatial arrangement of mouse TMEM217, SLC9C1, and sAC. The catalytic C1-C2 domains of sAC and the cyclic nucleotide-binding domain (CNBD) of SLC9C1 are adjacent to each other.

Table S1. Mass spectrometry results of SLC9 family proteins.

	Normalized total spectra (FLAG-IP)				Log2 protein quantities (mature spermatozoa)					
	Tmem217-/-; Tg				WT			*Tmem217*-/-		
Protein name	#1	#2	#3	WT	#1	#2	#3	#1	#2	#3
SLC9A1 (NHE1)	0.0	0.0	0.0	0.0	13.0	16.0	0.0	0.0	13.3	0.0
SLC9A2 (NHE2)	0.0	0.0	0.0	0.0	0.0	0.0	0.0	0.0	0.0	0.0
SLC9A3 (NHE3)	0.0	0.0	0.0	0.0	11.6	13.4	0.0	11.1	13.1	12.8
SLC9A4 (NHE4)	0.0	0.0	0.0	0.0	0.0	0.0	0.0	0.0	0.0	0.0
SLC9A5 (NHE5)	0.0	0.0	0.0	0.0	0.0	0.0	0.0	0.0	0.0	0.0
SLC9A6 (NHE6)	0.0	0.0	0.0	0.0	15.4	15.8	0.0	0.0	13.7	13.2
SLC9A7 (NHE7)	0.0	0.0	0.0	0.0	0.0	0.0	0.0	0.0	0.0	0.0
SLC9A8 (NHE8)	0.0	0.0	0.0	0.0	0.0	0.0	0.0	0.0	0.0	0.0
SLC9A9 (NHE9)	0.0	0.0	0.0	0.0	0.0	0.0	0.0	0.0	0.0	0.0
SLC9B1 (NHA1)	0.0	0.0	0.0	0.0	23.8	20.1	20.9	20.8	20.8	21.8
SLC9B2 (NHA2)	0.0	0.0	0.0	0.0	0.0	14.8	0.0	0.0	0.0	0.0
SLC9C1 (sNHE)	8.0	18.3	22.1	0.0	11.0	17.6	13.2	0.0	0.0	0.0

Quantitative values of SLC9 family proteins, identified via LC-MS analysis after FLAG immunoprecipitation (FLAG-IP) of WT and *Tmem217* KO; Tg testes (#1–#3) from Dataset S1 (left) or after preparing lysates from WT and *Tmem217* KO mature spermatozoa (Dataset S3) (right).

Table S2. Primers for RT-PCR, cloning, and genotyping.

Purpose	Gene	Sequence	Thermal cycling condition	Amplicon size (bp)
RT-PCR	mouse *Tmem217*	5'-AAAAGCTTGCCGCCATGAAGCATCACAGCTGG-3' and 5'-AAGCTAGCTTCACTGTGGTGCTGGACA-3'	94 °C 30s<br>60 °C 30s<br>72 °C 30s	589
RT-PCR	mouse *Actb*	5'-CATCCGTAAAGACCTCTATGCCAAC-3' and 5'-ATGGAGCCACCGATCCACA-3'	94 °C 30s<br>60 °C 30s<br>72 °C 30s	171
RT-PCR	human *TMEM217*	5'-AAGGATCCGCCGCCATGAAACAGCAGCAGTGGTGTGGG-3' and 5'-AAAAGCTTTTTTATACTTGTTTTACCAGAGCACC-3'	94 °C 30s<br>60 °C 30s<br>72 °C 30s	643
RT-PCR	human *ACTB*	5'-TGGCACCCAGCACAATGAA-3' and 5'-CTAAGTCATAGTCCGCCTAGAAGCA-3'	94 °C 30s<br>60 °C 30s<br>72 °C 30s	186
Cloning	Mouse *Slc9c1*	5'-AAGTCGACGCCGCCATGGAAATGGAAGAAATTTC-3' and 5'-AAGCTAGCGATGTTAATATTCTCTTCTATC-3'	94 °C 30s<br>60 °C 30s<br>72 °C 4min	3547
Cloning	Mouse *Slc9c1* (588-1175)	5'-AAGTCGACGCCGCCATGGAAAGCAAAAAACTCCTCTCC-3' and 5'-AAGCTAGCGATGTTAATATTCTCTTCTATC-3'	94 °C 30s<br>60 °C 30s<br>72 °C 2min	1783
Cloning	Mouse *Slc9c1* (733-1175)	5'-AAGTCGACGCCGCCATGAACGTGATTCGACTCCTTCG-3' and 5'-AAGCTAGCGATGTTAATATTCTCTTCTATC-3'	94 °C 30s<br>60 °C 30s<br>72 °C 2min	1348
Cloning	Mouse *Slc9c1* (1026-1175)	5'-AAGTCGACGCCGCCATGATTACTGCCCAAAAGATCCGAG-3' and 5'-AAGCTAGCGATGTTAATATTCTCTTCTATC-3'	94 °C 30s<br>60 °C 30s<br>72 °C 2min	469
Cloning	human *SLC9C1*	5'-AATCTAGAGCCGCCATGGCCGGCATCTTCAAGGAG-3' and 5'-AAGAATTCCTCCTTGCGCACCTTCCTCAGG-3'	94 °C 30s<br>60 °C 30s<br>72 °C 4min	3553
Genotyping	*Tmem217* WT (F1, R1)	5'-ATCCCCACCCCTGTCCAATA-3' and 5'-CTGGAAGTTCTCCGTGCAGT-3'	94 °C 30s<br>60 °C 30s<br>72 °C 60s	304
Genotyping	*Tmem217* KO (F2, R2)	5'-TTTGAATTCACTCGATATGG-3' and 5'-GCTGCCTTTGGATTAGGAGG-3'	94 °C 30s<br>60 °C 30s<br>72 °C 60s	KO:1229<br>WT:2300
Genotyping	*Clgn-Tmem217-3xFLAG* transgene	5'-TTGAGCGGGCCGCTTGCGCACTGG-3' and 5'-ACGTGAAGAGGACGATTTGG-3'	94 °C 30s<br>60 °C 30s<br>72 °C 60s	505

Movie S1. Sperm motility of WT mice at 10 min.
Sperm motility was videotaped at 50 frames per second 10 min after incubation. The movie is played at 20 frames/second (1/2.5 speed).

Movie S2. Sperm motility of WT mice at 120 min.
Sperm motility was videotaped at 50 frames per second 120 min after incubation. The movie is played at 20 frames/second (1/2.5 speed).

Movie S3. Sperm motility of *Tmem217* KO mice at 10 min.
Sperm motility was videotaped at 50 frames per second 10 min after incubation. The movie is played at 20 frames/second (1/2.5 speed).

Movie S4. Sperm motility of *Tmem217* KO mice at 120 min.
Sperm motility was videotaped at 50 frames per second 120 min after incubation. The movie is played at 20 frames/second (1/2.5 speed).

Movie S5. Sperm motility of *Tmem217* KO mice at 10 min with dbcAMP and IBMX.
Sperm motility was videotaped at 50 frames per second 10 min after incubation with dbcAMP and IBMX. The movie is played at 20 frames/second (1/2.5 speed).

Movie S6. Sperm motility of *Tmem217* KO mice at 120 min with dbcAMP and IBMX.
Sperm motility was videotaped at 50 frames per second 120 min after incubation with dbcAMP and IBMX. The movie is played at 20 frames/second (1/2.5 speed).

Dataset S1. TMEM217-interacting proteins identified via nanoLC-MS/MS analysis.

Dataset S2. Enriched Gene Ontology (GO) terms of TMEM217 interactors in testes.

Dataset S3. Proteins detected by nanoLC-MS/MS analysis of mature spermatozoa.

SI References

1. K. Tokuhiro, M. Ikawa, A. M. Benham, M. Okabe, Protein disulfide isomerase homolog PDILT is required for quality control of sperm membrane protein ADAM3 and male fertility [corrected]. *Proc Natl Acad Sci U S A* **109**, 3850-3855 (2012).
2. H. Miyata *et al.*, SPATA33 localizes calcineurin to the mitochondria and regulates sperm motility in mice. *Proc Natl Acad Sci U S A* **118** (2021).
3. Y. Muro *et al.*, Behavior of Mouse Spermatozoa in the Female Reproductive Tract from Soon after Mating to the Beginning of Fertilization. *Biol Reprod* **94**, 80 (2016).
4. E. Wertheimer *et al.*, Compartmentalization of distinct cAMP signaling pathways in mammalian sperm. *J Biol Chem* **288**, 35307-35320 (2013).
5. H. Miyata *et al.*, Testis-enriched kinesin KIF9 is important for progressive motility in mouse spermatozoa. *FASEB J* **34**, 5389-5400 (2020).
6. V. Gaysinskaya, I. Y. Soh, G. W. van der Heijden, A. Bortvin, Optimized flow cytometry isolation of murine spermatocytes. *Cytometry A* **85**, 556-565 (2014).
7. J. Kim *et al.*, SPATC1L maintains the integrity of the sperm head-tail junction. *EMBO Rep* **19** (2018).
8. B. M. Phelps, D. E. Koppel, P. Primakoff, D. G. Myles, Evidence that proteolysis of the surface is an initial step in the mechanism of formation of sperm cell surface domains. *J Cell Biol* **111**, 1839-1847 (1990).
9. G. Tiscornia, O. Singer, I. M. Verma, Production and purification of lentiviral vectors. *Nat Protoc* **1**, 241-245 (2006).
10. A. Morohoshi *et al.*, Nexin-Dynein regulatory complex component DRC7 but not FBXL13 is required for sperm flagellum formation and male fertility in mice. *PLoS Genet* **16**, e1008585 (2020).
11. Y. Lu *et al.*, 1700029I15Rik orchestrates the biosynthesis of acrosomal membrane proteins required for sperm-egg interaction. *Proc Natl Acad Sci U S A* **120**, e2207263120 (2023).
12. G. Yu, L. G. Wang, Y. Han, Q. Y. He, clusterProfiler: an R package for comparing biological themes among gene clusters. *OMICS* **16**, 284-287 (2012).
13. V. Demichev, C. B. Messner, S. I. Vernardis, K. S. Lilley, M. Ralser, DIA-NN: neural networks and interference correction enable deep proteome coverage in high throughput. *Nat Methods* **17**, 41-44 (2020).
14. J. Cox *et al.*, Accurate proteome-wide label-free quantification by delayed normalization and maximal peptide ratio extraction, termed MaxLFQ. *Mol Cell Proteomics* **13**, 2513-2526 (2014).
15. R. Kolde, Pheatmap: pretty heatmaps. *R package version* **1**, 726 (2019).
16. T. Tsaban *et al.*, CladeOScope: functional interactions through the prism of clade-wise co-evolution. *NAR Genom Bioinform* **3**, lqab024 (2021).
17. V. Kalienkova, M. F. Peter, J. Rheinberger, C. Paulino, Structures of a sperm-specific solute carrier gated by voltage and cAMP. *Nature* **623**, 202-209 (2023).
18. B. P. Hermann *et al.*, The Mammalian Spermatogenesis Single-Cell Transcriptome, from Spermatogonial Stem Cells to Spermatids. *Cell Rep* **25**, 1650-1667 e1658 (2018).

19. J. Abramson *et al.*, Addendum: Accurate structure prediction of biomolecular interactions with AlphaFold 3. *Nature* **636**, E4 (2024).
20. E. C. Meng *et al.*, UCSF ChimeraX: Tools for structure building and analysis. *Protein Sci* **32**, e4792 (2023).
